# Haematological consequences of acute uncomplicated falciparum malaria: a WorldWide Antimalarial Resistance Network pooled analysis of individual patient data

**DOI:** 10.1186/s12916-022-02265-9

**Published:** 2022-03-07

**Authors:** The WorldWide Antimalarial Resistance Network Falciparum Haematology Study Group, Rashid Mansoor, Robert J. Commons, Nicholas M. Douglas, Benjamin Abuaku, Jane Achan, Ishag Adam, George O. Adjei, Martin Adjuik, Bereket H. Alemayehu, Richard Allan, Elizabeth N. Allen, Anupkumar R. Anvikar, Emmanuel Arinaitwe, Elizabeth A. Ashley, Hazel Ashurst, Puji B. S. Asih, Nathan Bakyaita, Hubert Barennes, Karen I. Barnes, Leonardo Basco, Quique Bassat, Elisabeth Baudin, David J Bell, Delia Bethell, Anders Bjorkman, Caroline Boulton, Teun Bousema, Philippe Brasseur, Hasifa Bukirwa, Rebekah Burrow, Verena I. Carrara, Michel Cot, Umberto D’Alessandro, Debashish Das, Sabyasachi Das, Timothy M. E. Davis, Meghna Desai, Abdoulaye A. Djimde, Arjen M. Dondorp, Grant Dorsey, Chris J. Drakeley, Stephan Duparc, Emmanuelle Espié, Jean-Francois Etard, Catherine Falade, Jean Francois Faucher, Scott Filler, Carole Fogg, Mark Fukuda, Oumar Gaye, Blaise Genton, Awab Ghulam Rahim, Julius Gilayeneh, Raquel Gonzalez, Rebecca F. Grais, Francesco Grandesso, Brian Greenwood, Anastasia Grivoyannis, Christoph Hatz, Eva Maria Hodel, Georgina S. Humphreys, Jimee Hwang, Deus Ishengoma, Elizabeth Juma, S. Patrick Kachur, Piet A. Kager, Erasmus Kamugisha, Moses R. Kamya, Corine Karema, Kassoum Kayentao, Adama Kazienga, Jean-René Kiechel, Poul-Erik Kofoed, Kwadwo Koram, Peter G. Kremsner, David G. Lalloo, Moses Laman, Sue J. Lee, Bertrand Lell, Amelia W. Maiga, Andreas Mårtensson, Mayfong Mayxay, Wilfred Mbacham, Rose McGready, Hervé Menan, Didier Ménard, Frank Mockenhaupt, Brioni R. Moore, Olaf Müller, Alain Nahum, Jean-Louis Ndiaye, Paul N. Newton, Billy E. Ngasala, Frederic Nikiema, Akindeh M. Nji, Harald Noedl, Francois Nosten, Bernhards R. Ogutu, Olusola Ojurongbe, Lyda Osorio, Jean-Bosco Ouédraogo, Seth Owusu-Agyei, Anil Pareek, Louis K. Penali, Patrice Piola, Mateusz Plucinski, Zul Premji, Michael Ramharter, Caitlin L. Richmond, Lars Rombo, Cally Roper, Philip J. Rosenthal, Sam Salman, Albert Same-Ekobo, Carol Sibley, Sodiomon B. Sirima, Frank M. Smithuis, Fabrice A. Somé, Sarah G. Staedke, Peter Starzengruber, Nathalie Strub-Wourgaft, Inge Sutanto, Todd D. Swarthout, Din Syafruddin, Ambrose O. Talisuna, Walter R. Taylor, Emmanuel A. Temu, Julie I. Thwing, Halidou Tinto, Emiliana Tjitra, Offianan A. Touré, T. Hien Tran, Johan Ursing, Innocent Valea, Giovanni Valentini, Michele van Vugt, Lorenz von Seidlein, Stephen A. Ward, Vincent Were, Nicholas J. White, Charles J. Woodrow, William Yavo, Adoke Yeka, Issaka Zongo, Julie A. Simpson, Philippe J. Guerin, Kasia Stepniewska, Ric N. Price

**Affiliations:** Centre for Tropical Medicine and Global Health, Nuffield Department of Clinical Medicine, WorldWide Antimalarial Resistance Network (WWARN), University of Oxford, Oxford, UK

**Keywords:** *Plasmodium falciparum*, Artemisinin-based therapy, Non-artemisinin-based therapy, Antimalarials, Haemoglobin, Severe anaemia, Pooled analysis of individual patient data

## Abstract

**Background:**

*Plasmodium falciparum* malaria is associated with anaemia-related morbidity, attributable to host, parasite and drug factors. We quantified the haematological response following treatment of uncomplicated *P. falciparum* malaria to identify the factors associated with malarial anaemia.

**Methods:**

Individual patient data from eligible antimalarial efficacy studies of uncomplicated *P. falciparum* malaria, available through the WorldWide Antimalarial Resistance Network data repository prior to August 2015, were pooled using standardised methodology. The haematological response over time was quantified using a multivariable linear mixed effects model with nonlinear terms for time, and the model was then used to estimate the mean haemoglobin at day of nadir and day 7. Multivariable logistic regression quantified risk factors for moderately severe anaemia (haemoglobin < 7 g/dL) at day 0, day 3 and day 7 as well as a fractional fall ≥ 25% at day 3 and day 7.

**Results:**

A total of 70,226 patients, recruited into 200 studies between 1991 and 2013, were included in the analysis: 50,859 (72.4%) enrolled in Africa, 18,451 (26.3%) in Asia and 916 (1.3%) in South America. The median haemoglobin concentration at presentation was 9.9 g/dL (range 5.0–19.7 g/dL) in Africa, 11.6 g/dL (range 5.0–20.0 g/dL) in Asia and 12.3 g/dL (range 6.9–17.9 g/dL) in South America. Moderately severe anaemia (Hb < 7g/dl) was present in 8.4% (4284/50,859) of patients from Africa, 3.3% (606/18,451) from Asia and 0.1% (1/916) from South America. The nadir haemoglobin occurred on day 2 post treatment with a mean fall from baseline of 0.57 g/dL in Africa and 1.13 g/dL in Asia. Independent risk factors for moderately severe anaemia on day 7, in both Africa and Asia, included moderately severe anaemia at baseline (adjusted odds ratio (AOR) = 16.10 and AOR = 23.00, respectively), young age (age < 1 compared to ≥ 12 years AOR = 12.81 and AOR = 6.79, respectively), high parasitaemia (AOR = 1.78 and AOR = 1.58, respectively) and delayed parasite clearance (AOR = 2.44 and AOR = 2.59, respectively). In Asia, patients treated with an artemisinin-based regimen were at significantly greater risk of moderately severe anaemia on day 7 compared to those treated with a non-artemisinin-based regimen (AOR = 2.06 [95%CI 1.39–3.05], *p* < 0.001).

**Conclusions:**

In patients with uncomplicated *P. falciparum* malaria, the nadir haemoglobin occurs 2 days after starting treatment. Although artemisinin-based treatments increase the rate of parasite clearance, in Asia they are associated with a greater risk of anaemia during recovery.

**Supplementary Information:**

The online version contains supplementary material available at 10.1186/s12916-022-02265-9.

## Background

Malaria remains a major cause of anaemia in malaria endemic countries, with a complex pathogenesis attributable to red cell destruction and haematopoietic suppression [1] that can be compounded by malnutrition, helminth carriage and inherited blood disorders [2]. Artemisinin-based combination therapy (ACT) is the first-line antimalarial treatment for uncomplicated malaria in almost all endemic countries [3], achieving high cure rates, rapid parasite clearance and reduced ongoing transmission of the parasite [4, 5]. However, artemisinin derivatives can suppress reticulocytosis and contribute to haemolysis; their use has been associated with delayed-onset anaemia [6, 7]. The haematological recovery and adverse consequences of the artemisinin derivatives, following the treatment of falciparum malaria, may vary between different ACTs [8].

To assess the comparative benefits of different antimalarial treatment regimens, it is critical to quantify the haematological impact attributable to *P. falciparum* infection and the clinical and demographic factors that underlie this. The aim of this study was to determine the pattern of haematological recovery following uncomplicated falciparum malaria and define the risk factors for moderately severe haematological outcomes at baseline and during early follow-up.

## Methods

### The WWARN repository and study selection

Haemoglobin concentrations are often not reported in antimalarial trial publications, even if these data are collected. Since a review of published literature would not provide sufficiently comprehensive information, the focus of this individual patient data meta-analysis was on studies identified in the WWARN repository. The WWARN repository contains data from 451 antimalarial efficacy studies in which patients were enrolled from locations in 69 countries, with a diverse range of *P. falciparum* transmission intensities. Data in the repository have been standardised and collated using methodology described previously in the WWARN Clinical Module Data Management and Statistical Analysis Plan [9].

The WWARN repository was searched in August 2015 for all antimalarial efficacy studies of uncomplicated *P. falciparum* malaria in non-pregnant patients that followed subjects prospectively for a minimum of 28 days and reported haemoglobin concentration (or haematocrit) at least at baseline (day 0). Investigators of the identified studies were invited to participate in this study group and information was made available on the WWARN website [10]. Uncomplicated *P. falciparum* malaria was defined as microscopy-proven falciparum malaria without features of severe malaria [11]. Patients were excluded if they had severe malaria.

### Outcomes of interest

The primary outcome of the analysis was the risk of moderately severe anaemia (Hb < 7 g/dL) on day 7 after initiation of treatment. Secondary outcomes included the mean fall in haemoglobin at day of nadir and day 7, the timing of nadir haemoglobin, risk of moderately severe anaemia at days 0 and 3, and the risk of a large reduction in haemoglobin from baseline, defined as a fractional fall in Hb of ≥ 25% on day 3 or 7.

### Statistical methods

All statistical analyses were done using R (Version 3.2.5, The R Foundation for Statistical Computing) or Stata MP 15, based on an a priori statistical plan shared with data contributors [10].

Haematocrit measurements were converted to haemoglobin concentrations using the following formula: Haemoglobin = (Haematocrit − 5.62)/2.60 [12]. The timing of sampling was defined as day 0 if occurring on the day of enrolment / first day of treatment, with sequential numbering thereafter. Data were stratified by region (Africa, Asia and South America). Univariable and multivariable mixed effects logistic regression models were used to model risk of (i) moderately severe anaemia on day 0, 3 or 7 and (ii) large reduction in haemoglobin on day 3 or 7. Study site (sites within countries) was included as a random intercept in these models.

Changes in mean haemoglobin over time were examined, after stratifying by region, using linear mixed effects models. Fractional polynomial terms for time were fitted as random effects for patients to capture the nonlinear associations and random intercepts for patients and study site. All available haemoglobin measurements were included in these analyses. Additional analyses of mean haemoglobin over time within each region were undertaken, stratified by age group (< 5 years and ≥ 5 years).

For all regression models, independent risk factors were identified following the strategy recommended by Collet [13]. Covariates examined included the following: age in years (categorised as < 1 year, 1 to 4 years, 5 to 11 years and ≥ 12 years), sex, fever (temperature > 37.5 °C) on enrolment, baseline parasitaemia (after log transformation), mixed *Plasmodium* species infection, underweight (defined as weight-for-age *Z*-score <−2 for children younger than 5 years) [14], high parasitaemia (defined as > 100,000 parasites/μL [15]), presence of gametocytaemia on enrolment, transmission intensity, treatment (artemisinin-based therapy versus non-artemisinin-based therapy) and parasite clearance (early clearance on day 1 or day 2 versus delayed parasite clearance on day 3 or later). Red cell indices were not available. Malaria transmission intensity was defined based on estimates of *P. falciparum* prevalence rate (PfPR) according to enrolment year and location [16], assuming low transmission for study sites with a PfPR < 0.15, moderate transmission if PfPR 0.15 to < 0.40 and high transmission if PfPR ≥ 0.40. Fractional polynomials were used to define the nonlinear relationships between outcome and continuous covariates.

### Ethics

All data included in this analysis were obtained in accordance with ethical approvals from the countries of origin. The data are fully anonymised and cannot be traced back to individuals. This analysis did not require separate ethical approval according to the guidelines of the Oxford Central University Research Ethics Committee.

## Results

**Fig. 1:**
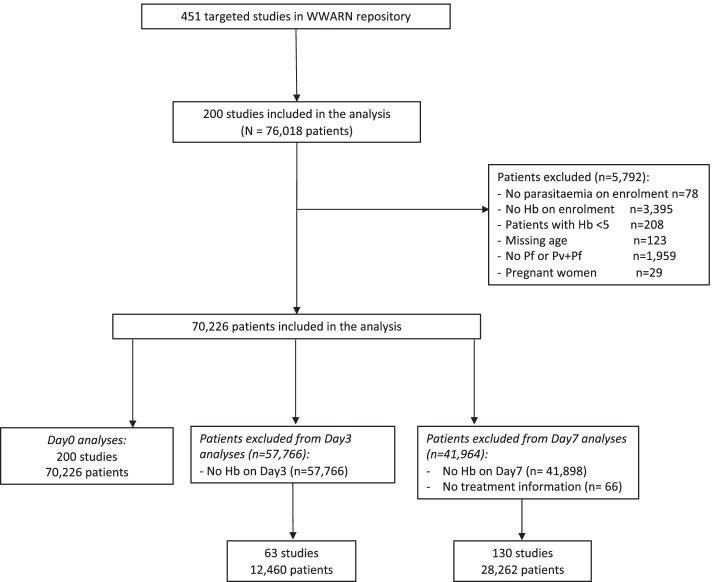
Study flow diagram. Hb—haemoglobin; Pf—*P. falciparum*; Pv—*P. vivax*

A total of 200 *P. falciparum* clinical trials undertaken between 1991 and 2013 met the inclusion criteria and were available for analysis (Fig. 1, Additional file 1: Tables S1-S2, and Additional file 2: Figure S1) [1, 17–177]. Individual records were available from 70,226 patients, of whom 50,859 (72.4%) were enrolled in Africa, 18,451 (26.3%) in Asia and 916 (1.3%) in South America (Table 1). In Asia and South America, 61.8% (11,963/19,367) of the patients were male and 35.0% (6775/19,357) were younger than 12 years old. In African studies, there was an equal sex distribution (51.1% males, 25,566/49,998) and 88.3% (44,890/50,859) were younger than 12 years old. Overall, 76.5% (53,730) of patients received artemisinin-based treatment varying from 72.5% in Africa, to 87.2% in Asia and 89.6% in South America (Table 1 and Additional file 3: Table S3). In the 53,730 patients receiving an artemisinin-based treatment, artemether-lumefantrine (AL) was administered in 34.2% (18,359) of cases, artesunate-amodiaquine (ASAQ) in 19.6% (10,536), artesunate-mefloquine (ASMQ) in 14.5% (7764), dihydroartemisinin-piperaquine (DP) in 15.3% (8197), and other artemisinin-based treatments in 16.5% (8874) (Additional file 3: Table S3).

**Table 1:** Demographic and baseline characteristics

	Africa	Asia	South America
	***N*** (%) or median (range)	***N*** (%) or median (range)	***N*** (%) or median (range)
Number of patients evaluated	50,859	18,451	916
Age (years)	3 (0.03–86.7)	16 (0.2–88.0)	23 (3.1–65.0)
*Age group*			
< 1 year	4562 (9.0)	67 (0.4)	0 (0)
1–4 years	31,225 (61.4)	2071 (11.2)	2 (0.2)
5–11 years	9103 (17.9)	4524 (24.5)	111 (12.1)
≥ 12 years	5969 (11.7)	11,789 (63.9)	803 (87.7)
*Sex*^a^			
Female	24,432 (48.9)	7054 (38.2)	350 (38.2)
Male	25,566 (51.1)	11,397 (61.8)	566 (61.8)
Haemoglobin (g/dL)^b^	9.9 (5.0–19.7)	11 (5.0–20.0)	12.3 (6.9–17.9)
Haematocrit (%)^c^	31.6 (10.0–54.0)	36.3 (14.4–55.0)	37.3 (18–54.1)
Derived haemoglobin (g/dL)^d^	9.9 (5.0–19.7)	11.6 (5.0–20.0)	12.3 (6.9–17.9)
*Anaemia*			
Moderately severe anaemia (haemoglobin < 7 g/dL)	4284 (8.4)	606 (3.3)	1 (0.1)
Moderate anaemia (haemoglobin 7–< 10 g/dL)	21,676 (42.6)	4260 (23.1)	78 (8.5)
No anaemia (haemoglobin ≥ 10 g/dL)	24,899 (49.0)	13,585 (73.6)	837 (91.4)
Temperature (°C)^e^	38 (34.0–42.0)	37.7 (34.0–42.0)	37.5 (35.1–42.0)
Fever (temperature > 37.5 °C)	32,266 (65.9)	8937 (54.2)	438 (48.0)
Parasitaemia (/μL)	21,600 (2.5–486,080)	9375 (7–499,712)	4490 (8–149,925)
High parasitaemia (> 100,000/μl)	5200 (10.2)	1548 (8.4)	3 (0.3)
Presence of gametocytaemia^f^	2339 (8.2)	1530 (11.2)	107 (11.8)
Underweight (WAZ < − 2)^g^	6205 (18.8)	781 (39.0)	2 (100)
*Species of infection*			
Mixed *P. falciparum* and *P. vivax*	0 (0)	1151 (6.2)	0 (0)
*P. falciparum* mono-infection	50,859 (100)	17,300 (93.8)	916 (100)
*Transmission setting*			
High	19,766 (38.9)	0 (0)	0 (0)
Moderate	15,357 (30.2)	561 (3.0)	0 (0)
Low	15,736 (30.9)	17,890 (97.0)	916 (100)
*Treatment*			
Artemisinin-based	36,823 (72.5)	16,086 (87.2)	821 (89.6)
Non-artemisinin-based	13,935 (27.5)	2360 (12.8)	95 (10.4)

Total number of patients enrolled in Africa was 50,859, Asia was 18,451 and South America was 916

^a^Data on patient sex were only available for 49,998 patients from Africa

^b^Data on baseline haemoglobin were only available for 47,778 patients from Africa and 7139 patients from Asia

^c^Data on baseline haematocrit were only available for 13,244 patients from Africa and 13,892 patients from Asia

^d^The following conversion from haematocrit was used: haemoglobin = (haematocrit − 5.62)/2.60

^e^Data on baseline temperature were only available for 48,982 patients from Africa and 16,483 patients from Asia

^f^Data on baseline gametocytes were only available for 28,453 patients from Africa, 13,697 patients from Asia and 904 patients from South America

^g^Data on weight-for-age *Z*-scores (WAZ) were only available for 33,048 patients from Africa, 2001 patients from Asia and 2 patients from South America. WAZ was only evaluated in children < 5 years

### Haematological status at enrolment

The haematological exclusion criteria differed between studies. The commonest haematological exclusion criterion was a haemoglobin < 5 g/dL (used in 126 studies where 39,940 patients were included), with 2 studies (483 patients) excluding patients with a haemoglobin < 6 g/dL, 9 studies (5964 patients) excluding patients with a haemoglobin < 7 g/dL and 3 studies (566 patients) excluding patients with a haemoglobin < 8 g/dL. In 60 studies, haematological exclusion criteria were not stated; Additional file 1: Table S2. There were 208 patients with a haemoglobin < 5 g/dL at baseline, who were excluded from further analysis, since they met the WHO criteria for severe malaria.

The median haemoglobin at enrolment was 9.9 g/dL (range 5.0–19.7 g/dL) in Africa, 11.6 g/dL (range 5.0–20.0 g/dL) in Asia and 12.3 g/dL (range 6.9–17.9 g/dL) in South America (Table 1). Moderately severe anaemia was defined as haemoglobin concentration < 7 g/dL and was present in 4891 (6.9%) patients at baseline, with a prevalence of 8.4% (4284/50,859) in Africa, 3.3% (606/18,451) in Asia and 0.1% (1/916) in South America. Owing to the limited numbers of patients from South America, all subsequent analyses were restricted to patients from either Africa or Asia and stratified by geographical location.

**Fig. 2:**
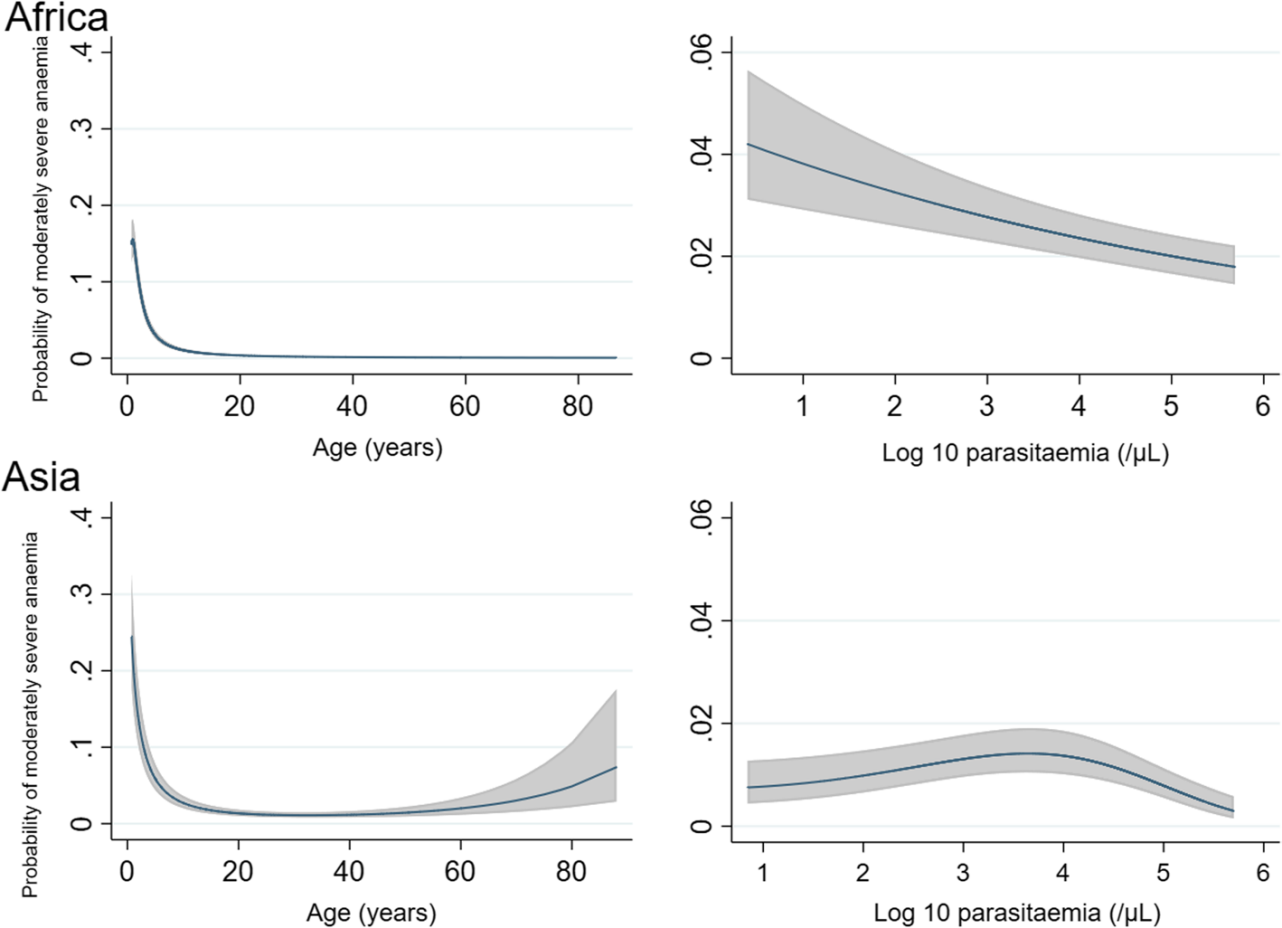
Relationship between predicted probabilities of moderately severe anaemia (haemoglobin < 7 g/dL) on enrolment and continuous covariates. Results are generated from the final multivariable models and are adjusted for mean values of other covariates (age, baseline parasitaemia, sex and fever). The model was restricted to children age > 0.75 years due to instability at the extremes of the data

The mean haemoglobin at enrolment varied with both age and baseline parasitaemia (Additional file 2: Figure S2). The main risk factors for moderately severe anaemia at baseline in both Africa and Asia were young age and presenting without high parasitaemia (> 100,000/μL); Additional file 3: Table S4 and S5. In Africa, the risk of moderately severe anaemia was inversely related to parasitaemia, whereas in Asia the risk rose to a peak at 10,000 parasites/μL, and decreased thereafter (Fig. 2).

### Haemoglobin profile following the start of treatment

**Fig. 3:**
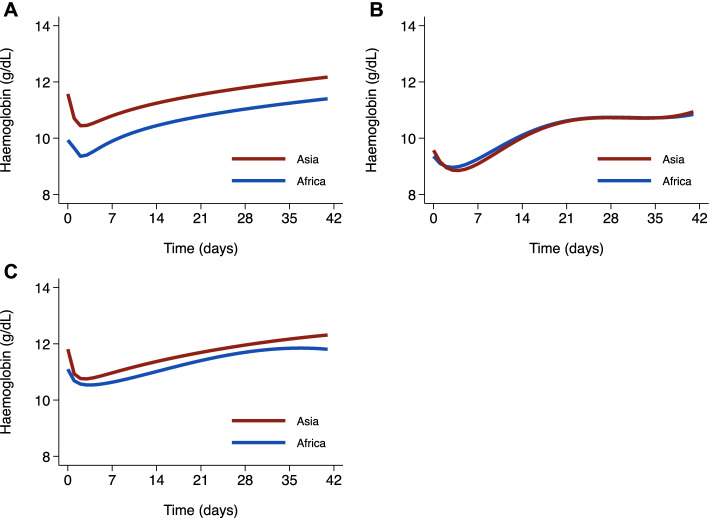
Relationship between haemoglobin and time from administration of first antimalarial dose for **A** all age groups, **B** patients < 5 years old and **C** patients ≥ 5 years old. Figure derived from linear mixed effects model with fractional polynomial terms for time

A linear mixed effects model of all haemoglobin concentrations over time showed that following the start of treatment haemoglobin concentration fell rapidly to a nadir on day 2 (Fig. 3). The estimated mean fall in haemoglobin in Africa was 0.57 g/dL at day 2 and 0.03 g/dL at day 7, with corresponding estimates in Asia of 1.13 g/dL and 0.78 g/dL. Haemoglobin concentrations returned to baseline by day 8 in Africa and day 22 in Asia, and thereafter continued to increase, reaching a mean concentration at day 42 of 11.40 g/dL (95%CI 11.28–11.52) in Africa (1.47 g/dL above baseline) and 12.17 g/dL (95%CI 11.98–12.36) in Asia (0.60 g/dL above baseline) (Fig. 3).

Haematological recovery was assumed to have occurred by day 42, and therefore, the observation on this day represented the baseline Hb of this patient population without infection. In African patients, 71.3% of the total fall in Hb from predicted baseline occurred before treatment and 28.3% after treatment. The corresponding percentages in Asia were 34.7% and 65.3% respectively.

Differences in the haemoglobin profiles between Africa and Asia were largely attributable to the variation in the age distributions of the study populations. When the analyses were stratified by age, the haemoglobin profiles were similar for the two continents (Fig. 3).

### Moderately severe anaemia after the start of treatment

Overall, 9.1% (1129/12,460) of patients had moderately severe anaemia (Hb < 7 g/dl) on day 3 and 4.4% (1241/28,262) on day 7. The risk of falling below 7 g/dL in people who did not have moderately severe anaemia at baseline was greater in Africa than in Asia on both day 3 (9.6% (987/10,278) versus 6.5% (142/2,182); *p* = 0.001) and day 7 (5.7% (987/17,198) versus 2.3% (254/11,064) respectively; *p* < 0.001).

**Fig. 4:**
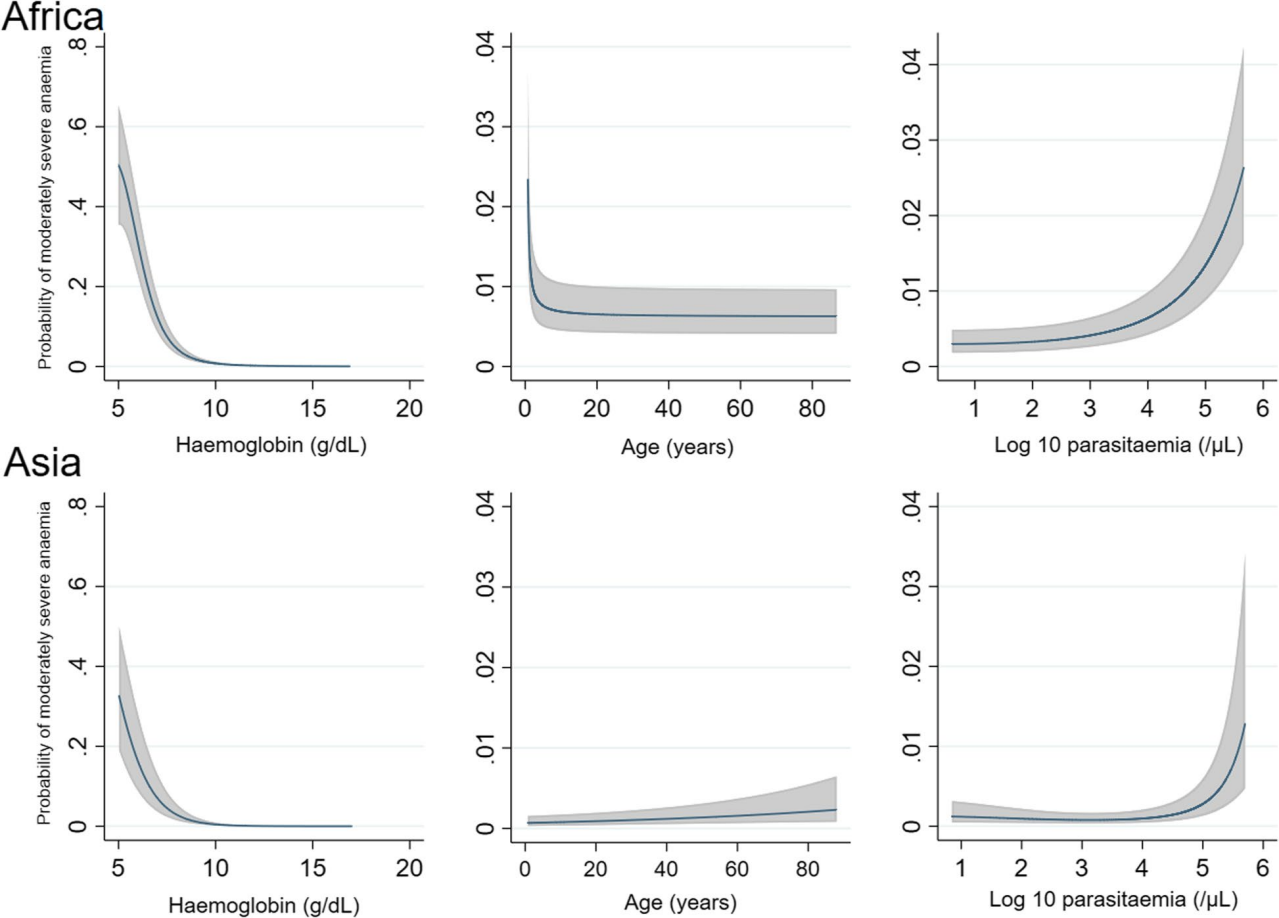
Relationship between predicted probabilities of moderately severe anaemia (haemoglobin < 7 g/dL) on day 7 and continuous covariates. Results come from the final multivariable models and are adjusted for mean values of other covariates (haemoglobin, age, parasitaemia, sex, fever, treatment (artemisinin-based vs non-artemisinin-based) and mixed infection (Asia only)). The model was restricted to children with age > 0.75 years and haemoglobin ≤ 17 g/dL due to instability at the extremes of the data

The following independent predictors of moderately severe anaemia at day 7 in both Africa and Asia were identified: moderately severe anaemia at baseline (AOR = 16.10 (95%CI 12.59–20.60), *p* < 0.001 for Africa and 23.00 (14.27–37.06), *p* < 0.001 for Asia), younger age (for age < 1 year compared to patients ≥ 12 years AOR = 12.81 (95%CI 6.79–24.17), *p* < 0.001 and AOR = 6.79 (95%CI 2.36–19.58), *p* < 0.001 and for age 1 to 4 years compared to patients ≥ 12 years AOR = 6.09 (95%CI 3.33–11.13), *p* < 0.001 and AOR = 2.87 (95%CI 1.84–4.47), *p* < 0.001) and parasitaemia > 100,000/μL (AOR = 1.78 (95%CI 1.42–2.24), *p* < 0.001 and 1.58 (1.10–2.27), *p* = 0.013) (Table 2 and Additional file 3: Table S6, Fig. 4). In Africa, fever (AOR = 1.66 (95%CI 1.33–2.09); *p* < 0.001) was an independent predictor of moderately severe anaemia whilst female sex (AOR = 0.80 (95%CI 0.69–0.93); *p* = 0.004) was protective. In Asia, female sex was an independent predictor (AOR = 1.51 (95%CI 1.15–1.99), *p* = 0.003) and mixed infection was protective (AOR = 0.44 (95%CI 0.24–0.80), *p* = 0.007). The effect of sex was explored by evaluating the models separately in children compared with adolescents and adults. In adolescents and adults (age ≥ 12 years), female sex was associated with moderately severe anaemia, and this reached statistical significance in Asia (AOR = 2.55 (95%CI 1.65–3.94), *p* < 0.001), but not Africa (AOR = 1.98 (95%CI 0.71–5.49), *p* = 0.191). In contrast, in children (age < 12 years) female sex was associated with a lower risk of moderately severe anaemia, reaching statistical significance in Africa (AOR = 0.78 (95%CI 0.67–0.91), *p* = 0.001), but not Asia (AOR = 0.98 (95%CI 0.73–1.32), *p* = 0.903).

**Table 2:** Risk factors for moderately severe anaemia (Hb < 7 g/dL) at day 7: Multivariable logistic regression

Parameter	Africa			Asia		
	% (Number with moderately severe anaemia/*N*)	AOR (95% CI)	*P* value	% (Number with moderately severe anaemia/*N*)	AOR (95% CI)	*P* value
*Age group*						
< 1 year	16.9% (177/1045)	12.81 (6.79–24.17)	< 0.001	9.7% (3/31)	6.79 (2.36–19.58)	< 0.001
1–4 years	6.5% (651/10,086)	6.09 (3.33–11.13)	< 0.001	7.3% (63/861)	2.87 (1.84–4.47)	< 0.001
5–11 years	3.6% (101/2826)	4.41 (2.30–8.48)	< 0.001	3.5% (83/2341)	2.35 (1.56–3.52)	< 0.001
≥ 12 years	0.7% (18/2706)	Reference		1.3% (91/6898)	Reference	
*Sex*						
Female	5.2% (415/8058)	0.80 (0.69–0.93)	0.004	3.2% (124/3884)	1.51 (1.15–1.99)	0.003
Male	6.2% (532/8605)	Reference		1.9% (116/6247)	Reference	
*Fever*						
Yes	7.0% (700/10,062)	1.66 (1.33–2.09)	< 0.001	2.7% (148/5578)	1.26 (0.92–1.71)	0.152
No	3.7% (247/6601)	Reference		2.0% (92/4553)	Reference	
*Moderately severe anaemia at day 0*						
Yes	42.4% (383/904)	16.10 (12.59–20.60)	< 0.001	32.6% (78/239)	23.00 (14.27–37.06)	< 0.001
No	3.6% (564/15,759)	Reference		1.6% (162/9892)		
*High parasitaemia*^a^						
Yes	9.4% (152/1621)	1.78 (1.42–2.24)	< 0.001	3.9% (35/900)	1.58 (1.10–2.27)	0.013
No	5.3% (795/15,042)	Reference		2.2% (205/9231)	Reference	
*Mixed infection*						
Yes	0% (0/0)			1.5% (11/717)	0.44 (0.24–0.80)	0.007
No	5.7% (947/16,663)			2.4% (229/9414)	Reference	
*Treatment*						
Artemisinin-based	5.5% (841/15,209)	1.01 (0.56–1.82)	0.987	2.5% (225/8958)	2.06 (1.39–3.05)	< 0.001
Non-artemisinin-based	7.3% (106/1454)	Reference		1.3% (15/1173)	Reference	

*N* total number of evaluable patients, *AOR* adjusted odds ratio

^a^Parasitaemia > 100,000/μL. Univariable risk factors are presented in Additional file 3: Table S6

Compared with those treated with non-artemisinin-based treatments, patients in Asia treated with artemisinin-based therapy were at significantly greater risk of moderately severe anaemia on day 7 (AOR = 2.06 (95%CI 1.39–3.05); *p* < 0.001), but this was not the case in African patients (AOR = 1.01 (95%CI 0.56–1.82); *p* = 0.987). In Asia, the difference in risk between artemisinin- and non-artemisinin-based treatments remained when only the 8570 patients enrolled before 2007 (when artemisinin resistance was first described in the Greater Mekong Subregion) were included in the model (AOR = 1.98 (95%CI 1.39–2.82); *p* < 0.001). Overall, the risk factors for moderately severe anaemia on day 3 were similar to those at day 7, with patients treated with artemisinin-based therapy at significantly greater risk of moderately severe anaemia on day 3 in Asia (AOR = 3.27 (95%CI 2.42–4.42); *p* < 0.001), but not in Africa (AOR = 0.69 (95%CI 0.32–1.49); *p* = 0.343) (Additional file 3: Table S7-S8 and Additional file 2: Figure S3).

The fractional fall in haemoglobin on day 7 was correlated positively with the baseline haemoglobin (*r* = 0.47; *p* < 0.001 adjusted for clustering of study site). A high baseline haemoglobin was associated with a greater risk of a large fractional fall (≥ 25%) on day 7 in Africa (AOR for every 1 g/dL increase in baseline haemoglobin = 1.52 (95%CI 1.40–1.65); *p* < 0.001) and in Asia (AOR for every 1 g/dL increase in baseline haemoglobin = 1.43 (95%CI 1.35–1.52); *p* < 0.001) (Additional file 2: Figure S4). Other risk factors for a large fractional fall in haemoglobin on day 7 were similar to the risk factors for moderately severe anaemia on this day (Additional file 3: Table S9).

### Parasite clearance and moderately severe anaemia

Of the 13,939 African patients who had haemoglobin concentrations measured on day 7, 11.1% (1547) were parasitaemic on day 2 and 2.6% (358) were parasitaemic on day 3. The corresponding proportions in Asia were 14.9% (1339/8960) and 4.2% (375/8960). After controlling for confounding factors, the risk of moderately severe anaemia at day 7 was greater in patients with delayed parasite clearance in both Africa (AOR = 2.44 (95%CI 1.59–3.75); *p* < 0.001) and Asia (AOR = 2.59 (95%CI 1.20–5.58); *p* = 0.015) (Table 3). There was no interaction between artemisinin use and delayed parasite clearance.

**Table 3:** Effect of parasite clearance time on moderately severe anaemia after treatment

	Africa			Asia		
	% (Number with moderately severe anaemia/*N*^a^)	AOR (95% CI)	*P* value	% (Number with moderately severe anaemia/*N*^a^)	AOR (95% CI)	*P* value
***Moderately severe anaemia on day 3***						
Clearance between day 0 and day 1	6.5% (221/3410)	Reference		4.4% (39/889)	Reference	
Clearance between day 1 and day 2	11.0% (473/4299)	1.52 (1.20–1.92)	0.001	6.5% (41/628)	1.16 (0.57–2.36)	0.685
Clearance between day 2 and day 3	15.0% (94/626)	2.26 (1.55–3.29)	< 0.001	6.3% (16/254)	2.04 (0.76–5.47)	0.156
Clearance after day 3	11.5% (9/78)	2.04 (0.82–5.09)	0.126	2.5% (5/197)	0.67 (0.06–7.08)	0.742
***Moderately severe anaemia on day 7***						
Clearance between day 0 and day 1	4.8% (243/5106)	Reference		1.6% (52/3177)	Reference	
Clearance between day 1 and day 2	6.1% (417/6834)	1.02 (0.76–1.35)	0.916	2.7% (100/3759)	1.32 (0.87–2.01)	0.187
Clearance between day 2 and day 3	8.4% (99/1182)	1.28 (0.88–1.85)	0.199	2.8% (26/938)	1.57 (0.92–2.68)	0.102
Clearance after day 3	12.4% (44/354)	2.44 (1.59–3.75)	< 0.001	2.9% (10/346)	2.59 (1.20–5.58)	0.015

Effects are adjusted for all independent predictors identified in the final multivariable models for Africa and Asia in Table 2 (Main text) and Additional file 3: Table S8

*AOR* adjusted odds ratio

^a^*N* number of patients for each variable/levels of factors

### Assessment of potential bias

Methodological factors potentially contributing to bias are presented in Additional file 1: Table S2. Although many studies were unblinded, haemoglobin measurement is automated, thus minimising the risk of observer bias. Publication bias was unlikely, since haemoglobin measurements were not a primary outcome in any of the publications and haemoglobin concentrations are unlikely to have influenced the decision to publish. Exclusion due to variable haemoglobin criteria will have caused a small reduction in the apparent proportion of patients with moderately severe anaemia at baseline and may also have artificially reduced the proportion of patients becoming severely anaemic during follow-up. In a sensitivity analysis, exclusion of patients from the 14 studies that had baseline haemoglobin cut-offs greater than 5 g/dL had minimal impact on the results (Additional file 3: Tables S10 and S11).

## Discussion

Our study provides a detailed analysis of haemoglobin concentration kinetics in patients with falciparum malaria, enrolled across geographically diverse regions. The available data, exceeding > 70,000 individual data from patients of all ages, provides unprecedented power to define the factors associated with the acute fall in haemoglobin before and after treatment. Malaria is due to an intraerythrocytic infection which results in a reduction of red blood cells, intra- and extravascular haemolysis, bone marrow suppression and sequestration [178]. The administration of antimalarial drugs inhibits these pathological processes by preventing parasite replication and limiting the duration of dyserythropoiesis. Hence, the haematological manifestations of malaria are a function of the duration and degree of parasitaemia prior to treatment and the speed of therapeutic response to antimalarial treatment. Our analysis demonstrated that in Africa, hence in generally relatively high transmission regions, approximately three quarters of the malaria-attributable fall in haemoglobin occurs before presentation and one quarter after treatment, whilst in Asia, in generally relatively low transmission settings, one third occurs before presentation and two-thirds after treatment. The relative drop in haemoglobin was positively correlated with baseline haemoglobin.

Although the greatest fall in haemoglobin occurred before treatment in Africa, our analysis focused primarily on factors associated with the subsequent fall and recovery which may be more amenable to clinical intervention. Consistent with a recent pooled analysis from Africa [179], our study found that in both Africa and Asia, the nadir haemoglobin occurred within 2 days of starting treatment and haemoglobin generally rose thereafter. Whilst a previous analysis identified that nadir haemoglobin occurred on day 7, this was based on weekly assessments, and thus would have missed the true nadir occurring between weekly observations [1]. In vulnerable populations, such as young children and pregnant women, who are at risk of adverse clinical outcomes, antimalarial clinical trials should implement a routine haemoglobin assessment at day 2 or 3 to ensure early diagnosis of severe anaemia.

The baseline haemoglobin in patients with falciparum malaria varied substantially with age and parasite density at presentation. After controlling for confounding factors, significant site to site variation remained, likely reflecting variations in transmission intensity, host immunity and factors unrelated to malaria. Patients from Asia tended to be older than those enrolled in Africa, but after controlling for age there were minimal differences in haemoglobin between regions, either at baseline or during follow-up. Following treatment, the absolute and proportional reductions in haemoglobin were greater in patients from Asia compared to Africa and were correlated with the higher baseline haemoglobin in Asian patients. Hence, patients presenting with a low haemoglobin concentration were less likely to experience a further fall in their haemoglobin.

The relationship between level of parasitaemia and degree of anaemia is complex [1, 179]. In Africa, anaemia at presentation was greatest in patients with low parasitaemias. There are several possible explanations for this. First, in highly endemic parts of Africa, robust immunity develops early, suppressing parasitaemia and symptoms. A substantial proportion of patients presenting with fever and low-level *Plasmodium* parasitaemia in these regions will have an alternative diagnosis, such as bacterial sepsis, which is also associated with anaemia [180]. Second, immune-mediated suppression of malaria symptoms can result in chronic, untreated parasitaemia that, over time, leads to significant suppression and dysregulation of haematopoiesis. Third, repeated episodes of malaria can result in splenic sequestration, with low-level peripheral parasitaemia, associated splenomegaly and dilutional anaemia [178, 181].

In Asia, the risk of anaemia at presentation increased with rising parasitaemia, peaking at 10,000 parasites/μL before decreasing thereafter. As transmission intensity in endemic parts of Asia is generally significantly lower than in Africa, immunity is less robust and a much greater proportion of infections will be symptomatic and of short duration. In this setting, anaemia will be related primarily to acute destruction of both parasitised and unparasitised red cells, the severity of which is correlated with the level of parasitaemia.

Treatment with artemisinin-based therapy in Asia was associated with a twofold higher risk of moderately severe anaemia (but not a large fractional fall in haemoglobin) within 7 days compared with non-artemisinin-based therapy, whereas in Africa, artemisinin-based treatment was not associated with an excess risk of early anaemia. This relationship was not attributable to the presence of artemisinin resistance. We hypothesise that rapid killing of intraerythrocytic parasites by artemisinins in non-immune Asian adults likely leads to more rapid clearance of whole red blood cells from the circulation than that occurring after slower acting drug treatments. In immune African patients, a greater proportion of infected red cells undergo targeted intraerythrocytic parasite removal (pitting) followed by a return to circulation, thus ameliorating the early development of anaemia [6]. Reticulocytosis probably also occurs more rapidly after treatment in immune compared with non-immune individuals [6]. Further studies are warranted to explore the differences in haematological response to treatment with artemisinin derivatives in populations with different levels of immunity. Our analysis is based upon studies conducted prior to 2014. In the last 5 years, artemisinin-resistant parasites have spread across the Greater Mekong Subregion [182], with recent reports confirming their presence in Sub-Saharan Africa [183, 184]. Slower parasite clearance times and subsequent emergence of resistance to partner drugs will ultimately lead to treatment failure that will impact the generally prompt haemoglobin recovery that we observed in our analysis.

Our study has a number of limitations. The analysis focused on the acute haematological impact of malaria and the early recovery phase and did not address the influence of late treatment failure on subsequent recovery to baseline haemoglobin concentrations. This will be addressed in a subsequent analysis. Our estimates of the pattern of haemoglobin changes during the first few days after diagnosis may have been influenced by selection bias, as only a small subset of patients had multiple haemoglobin measurements during the first 7 days of follow-up. Although we did not employ a traditional systematic review to identify eligible studies, our analysis is the largest meta-analysis to date of patients treated for malaria in both Africa and Asia. This unprecedented data collection ensures robust parameter estimates and minimises the risk of inclusion bias. Furthermore, a systematic review would not preclude bias, since some studies recorded haemoglobin/haematocrit measurements but did not present these data in published manuscripts. Whilst the results of the current study are likely to be generalisable to Africa and Asia, the small number of patients from the Americas prevents the generalisability of our findings to this region. An additional potential cause of bias is the exclusion of patients from the original studies, prior to pooling, according to variable definitions of severe anaemia. Almost two-thirds of studies excluded patients with a haemoglobin < 5 g/dL, with a few studies excluding patients based on higher cut-offs and the remaining 30% having an unknown cut-off. Additional limitations of our study include the use of various methodologies to measure haematocrit or haemoglobin, a lack of a robust conversion factor to adjust haematocrit to haemoglobin in different studies’ populations and no reliable data on the following confounding factors that can influence haemoglobin and its recovery: the duration of prior parasitaemia (which has been shown to correlate with anaemia at presentation [178]), host genetic factors associated with anaemia (e.g. sickle cell anaemia, thalassaemia), administration of haematinics (or treatment for anaemia) and hydration status.

## Conclusions

In conclusion, the majority of patients with uncomplicated falciparum malaria had a modest fall in haemoglobin following treatment, before subsequent improvement in haemoglobin during recovery. Despite highly effective treatment, some patients remained at significant risk of moderately severe anaemia. Young children had a particularly high risk, likely related to lower immunity and high initial peripheral parasitaemia. The risk of anaemia is exacerbated by prolonged parasitaemia prior to presentation [1] or delayed parasite clearance, both of which are associated with suboptimal treatment regimens particularly in areas where antimalarial drug resistance was emerging [185]. Whilst artemisinin-based treatment generally ensured rapid parasite clearance and high efficacy, in Asia their use was associated with a greater risk of moderately severe anaemia on day 3 and day 7 that could not be accounted for by an underlying rise in artemisinin resistance. Early diagnosis of malaria and treatment with highly effective antimalarials remains critical in minimising anaemia associated with *P. falciparum* infection.

## Supplementary Information

**Additional file 1: Table S1.** Describes studies included in the analysis. **Table S2.** Describes assessment of bias by included study.

**Additional file 2: Figure S1.** Describes study sites. **Figure S2.** Describes the relationship between haemoglobin on enrolment and continuous covariates. **Figure S3.** Describes the relationship between the predicted probability of moderately severe anaemia on day 3 and continuous covariates. **Figure S4.** Describes the relationship between the predicted probability of a large fractional fall in haemoglobin on day 7 and continuous covariates.

**Additional file 3: Table S3.** Describes the overview of antimalarial treatments. **Table S4.** Describes the risk factors for moderately severe anaemia at enrolment (univariable logistic regression). **Table S5.** Describes the risk factors for moderately severe anaemia at enrolment (multivariable logistic regression). **Table S6.** Describes the risk factors for moderately severe anaemia at day 7 (univariable logistic regression). **Table S7.** Describes the risk factors for moderately severe anaemia at day 3 (univariable logistic regression). **Table S8.** Describes the risk factors for moderately severe anaemia at day 3 (multivariable logistic regression). **Table S9.** Describes the risk factors for a large fractional fall in haemoglobin by day 7. **Table S10.** Describes the sensitivity analysis for risk factors for moderately severe anaemia at enrolment (multivariable logistic regression). **Table S11.** Describes the sensitivity analysis for risk factors for moderately severe anaemia at day 7 (multivariable logistic regression).

## Data Availability

The data that support the findings of this study are available for access via the WorldWide Antimalarial Resistance Network (WWARN.org). Requests for access will be reviewed by a Data Access Committee to ensure that use of data protects the interests of the participants and researchers according to the terms of ethics approval and principles of equitable data sharing. Requests can be submitted by email to malariaDAC@iddo.org via the Data Access Form available at WWARN.org/accessing-data. The WWARN platform is registered with the Registry of Research Data Repositories (re3data.org).

## Abbreviations

ACT: Artemisinin-based combination therapy
AL: Artemether-lumefantrine
AOR: Adjusted odds ratio
ASAQ: Artesunate-amodiaquine
ASMQ: Artesunate-mefloquine
CI: Confidence interval
DP: Dihydroartemisinin-piperaquine
PfPR: *P. falciparum* parasite rate
WAZ: Weight-for-age *Z*-score
WHO: World Health Organization

## References

[1] Price RN, Simpson JA, Nosten F, Luxemburger C, Hkirjaroen L, ter Kuile F, Chongsuphajaisiddhi T, White NJ (2001). Factors contributing to anemia after uncomplicated falciparum malaria. Am J Trop Med Hyg.

[2] Menendez C, Fleming AF, Alonso PL (2000). Malaria-related anaemia. Parasitol Today.

[3] World Health Organization (2017). World Malaria Report 2017.

[4] White NJ (1999). Delaying antimalarial drug resistance with combination chemotherapy. Parassitologia.

[5] Cao XT, Bethell DB, Pham TP, Ta TT, Tran TN, Nguyen TT, Pham TT, Nguyen TT, Day NP, White NJ (1997). Comparison of artemisinin suppositories, intramuscular artesunate and intravenous quinine for the treatment of severe childhood malaria. Trans R Soc Trop Med Hyg.

[6] Fanello C, Onyamboko M, Lee SJ, Woodrow C, Setaphan S, Chotivanich K, Buffet P, Jaureguiberry S, Rockett K, Stepniewska K (2017). Post-treatment haemolysis in African children with hyperparasitaemic falciparum malaria; a randomized comparison of artesunate and quinine. BMC Infect Dis.

[7] Plewes K, Haider MS, Kingston HW, Yeo TW, Ghose A, Hossain MA, Dondorp AM, Turner GD, Anstey NM (2015). Severe falciparum malaria treated with artesunate complicated by delayed onset haemolysis and acute kidney injury. Malar J.

[8] Zwang J, Ndiaye JL, Djimde A, Dorsey G, Martensson A, Karema C, Olliaro P (2012). Comparing changes in haematologic parameters occurring in patients included in randomized controlled trials of artesunate-amodiaquine vs single and combination treatments of uncomplicated falciparum in sub-Saharan Africa. Malar J.

[9] WorldWide Antimalarial Resistance Network (WWARN) (2012). WWARN Clinical Module: Data Management and Statistical Analysis Plan Version 1.2.

[10] A pooled analysis of haematological recovery after treatment with an ACT for Plasmodium falciparum Version 17.07.14 [http://www.wwarn.org/working-together/study-groups/haematology-study-group]

[11] World Health Organization. Guideline for the treatment of malaria. 3rd ed. World Health Organization. Geneva; 2015.

[12] Lee SJ, Stepniewska K, Anstey N, Ashley E, Barnes K, Binh TQ, D'Alessandro U, Day NP, de Vries PJ, Dorsey G (2008). The relationship between the haemoglobin concentration and the haematocrit in Plasmodium falciparum malaria. Malar J.

[13] Collett D (2015). Modelling survival data in medical research.

[14] World Health Organization (2006). WHO child growth standards: length/height for age, weight-for-age, weight-for-length, weight-for-height and body mass index-for-age, methods and development.

[15] World Health Organization (2014). Severe Malaria. Tropical Med Int Health.

[16] Weiss DJ, Lucas TCD, Nguyen M, Nandi AK, Bisanzio D, Battle KE, Cameron E, Twohig KA, Pfeffer DA, Rozier JA (2019). Mapping the global prevalence, incidence, and mortality of Plasmodium falciparum, 2000-17: a spatial and temporal modelling study. Lancet.

[17] Luxemburger C, Nosten F, Raimond SD, Chongsuphajaisiddhi T, White NJ (1995). Oral artesunate in the treatment of uncomplicated hyperparasitemic falciparum malaria. Am J Trop Med Hyg.

[18] Bouyou-Akotet MK, Ramharter M, Ngoungou EB, Mamfoumbi MM, Mihindou MP, Missinou MA, Kurth F, Belard S, Agnandji ST, Issifou S (2010). Efficacy and safety of a new pediatric artesunate-mefloquine drug formulation for the treatment of uncomplicated falciparum malaria in Gabon. Wien Klin Wochenschr.

[19] Menan H, Faye O, Same-Ekobo A, Oga AS, Faye B, Kiki Barro CP, Kuete T, N'Diaye JL, Vicky AM, Tine R (2011). Comparative study of the efficacy and tolerability of dihydroartemisinin-piperaquine-trimethoprim versus artemether-lumefantrine in the treatment of uncomplicated Plasmodium falciparum malaria in Cameroon, Ivory Coast and Senegal. Malar J.

[20] Depoortere E, Guthmann JP, Presse J, Sipilanyambe N, Nkandu E, Balkan S, de Pecoulas PE, Legros D (2005). Efficacy and effectiveness of the combination of sulfadoxine/pyrimethamine and a 3-day course of artesunate for the treatment of uncomplicated falciparum malaria in a refugee settlement in Zambia. Tropical Med Int Health.

[21] Checchi F, Roddy P, Kamara S, Williams A, Morineau G, Wurie AR, Hora B, Lamotte N, Baerwaldt T, Heinzelmann A (2005). Evidence basis for antimalarial policy change in Sierra Leone: five in vivo efficacy studies of chloroquine, sulphadoxine-pyrimethamine and amodiaquine. Tropical Med Int Health.

[22] Guthmann JP, Ampuero J, Fortes F, van Overmeir C, Gaboulaud V, Tobback S, Dunand J, Saraiva N, Gillet P, Franco J (2005). Antimalarial efficacy of chloroquine, amodiaquine, sulfadoxine-pyrimethamine, and the combinations of amodiaquine + artesunate and sulfadoxine-pyrimethamine + artesunate in Huambo and Bie provinces, central Angola. Trans R Soc Trop Med Hyg.

[23] Hien TT, Thuy-Nhien NT, Phu NH, Boni MF, Thanh NV, Nha-Ca NT, Thai le H, Thai CQ, Toi PV, Thuan PD (2012). In vivo susceptibility of Plasmodium falciparum to artesunate in Binh Phuoc Province, Vietnam. Malar J.

[24] Starzengruber P, Swoboda P, Fuehrer HP, Khan WA, Hofecker V, Siedl A, Fally M, Graf O, Teja-Isavadharm P, Haque R (2012). Current status of artemisinin-resistant falciparum malaria in South Asia: a randomized controlled artesunate monotherapy trial in Bangladesh. PLoS One.

[25] Swarthout TD, van den Broek IV, Kayembe G, Montgomery J, Pota H, Roper C (2006). Artesunate + amodiaquine and artesunate + sulphadoxine-pyrimethamine for treatment of uncomplicated malaria in Democratic Republic of Congo: a clinical trial with determination of sulphadoxine and pyrimethamine-resistant haplotypes. Tropical Med Int Health.

[26] Valea I, Tinto H, Traore-Coulibaly M, Toe LC, Lindegardh N, Tarning J, Van Geertruyden JP, D'Alessandro U, Davies GR, Ward SA (2014). Pharmacokinetics of co-formulated mefloquine and artesunate in pregnant and non-pregnant women with uncomplicated Plasmodium falciparum infection in Burkina Faso. J Antimicrob Chemother.

[27] Kayentao K, Maiga H, Newman RD, McMorrow ML, Hoppe A, Yattara O, Traore H, Kone Y, Guirou EA, Saye R (2009). Artemisinin-based combinations versus amodiaquine plus sulphadoxine-pyrimethamine for the treatment of uncomplicated malaria in Faladje, Mali. Malar J.

[28] Menard D, Ratsimbasoa A, Randrianarivelojosia M, Rabarijaona LP, Raharimalala L, Domarle O, Randrianasolo L, Randriamanantena A, Jahevitra M, Andriantsoanirina V (2008). Assessment of the efficacy of antimalarial drugs recommended by the National Malaria Control Programme in Madagascar: up-dated baseline data from randomized and multi-site clinical trials. Malar J.

[29] Tine RC, Faye B, Sylla K, Ndiaye JL, Ndiaye M, Sow D, Lo AC, Abiola A, Ba MC, Gaye O (2012). Efficacy and tolerability of a new formulation of artesunate-mefloquine for the treatment of uncomplicated malaria in adult in Senegal: open randomized trial. Malar J.

[30] Price RN, Nosten F, Luxemburger C, van Vugt M, Phaipun L, Chongsuphajaisiddhi T, White NJ (1997). Artesunate/mefloquine treatment of multi-drug resistant falciparum malaria. Trans R Soc Trop Med Hyg.

[31] Jullien V, Valecha N, Srivastava B, Sharma B, Kiechel JR (2014). Population pharmacokinetics of mefloquine, administered as a fixed-dose combination of artesunate-mefloquine in Indian patients for the treatment of acute uncomplicated Plasmodium falciparum malaria. Malar J.

[32] Piola P, Fogg C, Bajunirwe F, Biraro S, Grandesso F, Ruzagira E, Babigumira J, Kigozi I, Kiguli J, Kyomuhendo J (2005). Supervised versus unsupervised intake of six-dose artemether-lumefantrine for treatment of acute, uncomplicated Plasmodium falciparum malaria in Mbarara, Uganda: a randomised trial. Lancet.

[33] Smithuis F, Kyaw MK, Phe O, Win T, Aung PP, Oo AP, Naing AL, Nyo MY, Myint NZ, Imwong M (2010). Effectiveness of five artemisinin combination regimens with or without primaquine in uncomplicated falciparum malaria: an open-label randomised trial. Lancet Infect Dis.

[34] Price RN, Nosten F, Luxemburger C, Kham A, Brockman A, Chongsuphajaisiddhi T, White NJ (1995). Artesunate versus artemether in combination with mefloquine for the treatment of multidrug-resistant falciparum malaria. Trans R Soc Trop Med Hyg.

[35] Tekete MM, Toure S, Fredericks A, Beavogui AH, Sangare CP, Evans A, Smith P, Maiga H, Traore ZI, Doumbo OK (2011). Effects of amodiaquine and artesunate on sulphadoxine-pyrimethamine pharmacokinetic parameters in children under five in Mali. Malar J.

[36] Falade C, Makanga M, Premji Z, Ortmann CE, Stockmeyer M, de Palacios PI (2005). Efficacy and safety of artemether-lumefantrine (Coartem) tablets (six-dose regimen) in African infants and children with acute, uncomplicated falciparum malaria. Trans R Soc Trop Med Hyg.

[37] Arinaitwe E, Sandison TG, Wanzira H, Kakuru A, Homsy J, Kalamya J, Kamya MR, Vora N, Greenhouse B, Rosenthal PJ (2009). Artemether-lumefantrine versus dihydroartemisinin-piperaquine for falciparum malaria: a longitudinal, randomized trial in young Ugandan children. Clin Infect Dis.

[38] Dorsey G, Staedke S, Clark TD, Njama-Meya D, Nzarubara B, Maiteki-Sebuguzi C, Dokomajilar C, Kamya MR, Rosenthal PJ (2007). Combination therapy for uncomplicated falciparum malaria in Ugandan children: a randomized trial. JAMA.

[39] Tarning J, Zongo I, Some FA, Rouamba N, Parikh S, Rosenthal PJ, Hanpithakpong W, Jongrak N, Day NP, White NJ (2012). Population pharmacokinetics and pharmacodynamics of piperaquine in children with uncomplicated falciparum malaria. Clin Pharmacol Ther.

[40] Moore BR, Benjamin JM, Salman S, Griffin S, Ginny E, Page-Sharp M, Robinson LJ, Siba P, Batty KT, Mueller I (2014). Effect of coadministered fat on the tolerability, safety, and pharmacokinetic properties of dihydroartemisinin-piperaquine in Papua New Guinean children with uncomplicated malaria. Antimicrob Agents Chemother.

[41] Zongo I, Dorsey G, Rouamba N, Tinto H, Dokomajilar C, Guiguemde RT, Rosenthal PJ, Ouedraogo JB (2007). Artemether-lumefantrine versus amodiaquine plus sulfadoxine-pyrimethamine for uncomplicated falciparum malaria in Burkina Faso: a randomised non-inferiority trial. Lancet.

[42] Dondorp AM, Nosten F, Yi P, Das D, Phyo AP, Tarning J, Lwin KM, Ariey F, Hanpithakpong W, Lee SJ (2009). Artemisinin resistance in Plasmodium falciparum malaria. N Engl J Med.

[43] Faye B, Ndiaye JL, Ndiaye D, Dieng Y, Faye O, Gaye O (2007). Efficacy and tolerability of four antimalarial combinations in the treatment of uncomplicated Plasmodium falciparum malaria in Senegal. Malar J.

[44] Awab GR, Pukrittayakamee S, Imwong M, Dondorp AM, Woodrow CJ, Lee SJ, Day NP, Singhasivanon P, White NJ, Kaker F (2010). Dihydroartemisinin-piperaquine versus chloroquine to treat vivax malaria in Afghanistan: an open randomized, non-inferiority, trial. Malar J.

[45] Stivanello E, Cavailler P, Cassano F, Omar SA, Kariuki D, Mwangi J, Piola P, Guthmann JP (2004). Efficacy of chloroquine, sulphadoxine-pyrimethamine and amodiaquine for treatment of uncomplicated Plasmodium falciparum malaria in Kajo Keji county, Sudan. Tropical Med Int Health.

[46] Karema C, Fanello CI, van Overmeir C, van Geertruyden JP, van Doren W, Ngamije D, D'Alessandro U (2006). Safety and efficacy of dihydroartemisinin/piperaquine (Artekin) for the treatment of uncomplicated Plasmodium falciparum malaria in Rwandan children. Trans R Soc Trop Med Hyg.

[47] Abdulla S, Sagara I, Borrmann S, D'Alessandro U, Gonzalez R, Hamel M, Ogutu B, Martensson A, Lyimo J, Maiga H (2008). Efficacy and safety of artemether-lumefantrine dispersible tablets compared with crushed commercial tablets in African infants and children with uncomplicated malaria: a randomised, single-blind, multicentre trial. Lancet.

[48] Vugt MV, Wilairatana P, Gemperli B, Gathmann I, Phaipun L, Brockman A, Luxemburger C, White NJ, Nosten F, Looareesuwan S (1999). Efficacy of six doses of artemether-lumefantrine (benflumetol) in multidrug-resistant Plasmodium falciparum malaria. Am J Trop Med Hyg.

[49] Sylla K, Abiola A, Tine RC, Faye B, Sow D, Ndiaye JL, Ndiaye M, Lo AC, Folly K, Ndiaye LA (2013). Monitoring the efficacy and safety of three artemisinin based-combinations therapies in Senegal: results from two years surveillance. BMC Infect Dis.

[50] Allen EN, Little F, Camba T, Cassam Y, Raman J, Boulle A, Barnes KI (2009). Efficacy of sulphadoxine-pyrimethamine with or without artesunate for the treatment of uncomplicated Plasmodium falciparum malaria in southern Mozambique: a randomized controlled trial. Malar J.

[51] van Vugt M, Leonardi E, Phaipun L, Slight T, Thway KL, McGready R, Brockman A, Villegas L, Looareesuwan S, White NJ (2002). Treatment of uncomplicated multidrug-resistant falciparum malaria with artesunate-atovaquone-proguanil. Clin Infect Dis.

[52] Ashley EA, Krudsood S, Phaiphun L, Srivilairit S, McGready R, Leowattana W, Hutagalung R, Wilairatana P, Brockman A, Looareesuwan S (2004). Randomized, controlled dose-optimization studies of dihydroartemisinin-piperaquine for the treatment of uncomplicated multidrug-resistant falciparum malaria in Thailand. J Infect Dis.

[53] Price RN, Uhlemann AC, van Vugt M, Brockman A, Hutagalung R, Nair S, Nash D, Singhasivanon P, Anderson TJ, Krishna S (2006). Molecular and pharmacological determinants of the therapeutic response to artemether-lumefantrine in multidrug-resistant Plasmodium falciparum malaria. Clin Infect Dis.

[54] Yeka A, Banek K, Bakyaita N, Staedke SG, Kamya MR, Talisuna A, Kironde F, Nsobya SL, Kilian A, Slater M (2005). Artemisinin versus nonartemisinin combination therapy for uncomplicated malaria: randomized clinical trials from four sites in Uganda. PLoS Med.

[55] Yeka A, Tibenderana J, Achan J, D'Alessandro U, Talisuna AO (2013). Efficacy of quinine, artemether-lumefantrine and dihydroartemisinin-piperaquine as rescue treatment for uncomplicated malaria in Ugandan children. PLoS One.

[56] Guthmann JP, Kasparian S, Phetsouvanh R, Nathan N, Garcia M, Phompida S, Brockman A, Gastellu M, Legros D (2002). The efficacy of chloroquine for the treatment of acute, uncomplicated, Plasmodium falciparum malaria in Laos. Ann Trop Med Parasitol.

[57] Anvikar AR, Sharma B, Shahi BH, Tyagi PK, Bose TK, Sharma SK, Srivastava P, Srivastava B, Kiechel JR, Dash AP (2012). Artesunate-amodiaquine fixed dose combination for the treatment of Plasmodium falciparum malaria in India. Malar J.

[58] Das D, Tripura R, Phyo AP, Lwin KM, Tarning J, Lee SJ, Hanpithakpong W, Stepniewska K, Menard D, Ringwald P (2013). Effect of high-dose or split-dose artesunate on parasite clearance in artemisinin-resistant falciparum malaria. Clin Infect Dis.

[59] Schramm B, Valeh P, Baudin E, Mazinda CS, Smith R, Pinoges L, Sundaygar T, Zolia YM, Jones JJ, Comte E (2013). Tolerability and safety of artesunate-amodiaquine and artemether-lumefantrine fixed dose combinations for the treatment of uncomplicated Plasmodium falciparum malaria: two open-label, randomized trials in Nimba County, Liberia. Malar J.

[60] Agarwal A, McMorrow M, Onyango P, Otieno K, Odero C, Williamson J, Kariuki S, Kachur SP, Slutsker L, Desai M (2013). A randomized trial of artemether-lumefantrine and dihydroartemisinin-piperaquine in the treatment of uncomplicated malaria among children in western Kenya. Malar J.

[61] Grande T, Bernasconi A, Erhart A, Gamboa D, Casapia M, Delgado C, Torres K, Fanello C, Llanos-Cuentas A, D'Alessandro U (2007). A randomised controlled trial to assess the efficacy of dihydroartemisinin-piperaquine for the treatment of uncomplicated falciparum malaria in Peru. PLoS One.

[62] Adjuik M, Agnamey P, Babiker A, Borrmann S, Brasseur P, Cisse M, Cobelens F, Diallo S, Faucher JF, Garner P (2002). Amodiaquine-artesunate versus amodiaquine for uncomplicated Plasmodium falciparum malaria in African children: a randomised, multicentre trial. Lancet.

[63] Bakyaita N, Dorsey G, Yeka A, Banek K, Staedke SG, Kamya MR, Talisuna A, Kironde F, Nsobya S, Kilian A (2005). Sulfadoxine-pyrimethamine plus chloroquine or amodiaquine for uncomplicated falciparum malaria: a randomized, multisite trial to guide national policy in Uganda. Am J Trop Med Hyg.

[64] van Vugt M, Brockman A, Gemperli B, Luxemburger C, Gathmann I, Royce C, Slight T, Looareesuwan S, White NJ, Nosten F (1998). Randomized comparison of artemether-benflumetol and artesunate-mefloquine in treatment of multidrug-resistant falciparum malaria. Antimicrob Agents Chemother.

[65] Grandesso F, Hagerman A, Kamara S, Lam E, Checchi F, Balkan S, Scollo G, Durand R, Guthmann JP (2006). Low efficacy of the combination artesunate plus amodiaquine for uncomplicated falciparum malaria among children under 5 years in Kailahun, Sierra Leone. Tropical Med Int Health.

[66] Kamugisha E, Jing S, Minde M, Kataraihya J, Kongola G, Kironde F, Swedberg G (2012). Efficacy of artemether-lumefantrine in treatment of malaria among under-fives and prevalence of drug resistance markers in Igombe-Mwanza, north-western Tanzania. Malar J.

[67] Plucinski MM, Talundzic E, Morton L, Dimbu PR, Macaia AP, Fortes F, Goldman I, Lucchi N, Stennies G, MacArthur JR (2015). Efficacy of artemether-lumefantrine and dihydroartemisinin-piperaquine for treatment of uncomplicated malaria in children in Zaire and Uige Provinces, Angola. Antimicrob Agents Chemother.

[68] Nahum A, Erhart A, Gazard D, Agbowai C, Van Overmeir C, van Loen H, Menten J, Akogbeto M, Coosemans M, Massougbodji A (2007). Adding artesunate to sulphadoxine-pyrimethamine greatly improves the treatment efficacy in children with uncomplicated falciparum malaria on the coast of Benin, West Africa. Malar J.

[69] Coulibaly B, Pritsch M, Bountogo M, Meissner PE, Nebie E, Klose C, Kieser M, Berens-Riha N, Wieser A, Sirima SB (2015). Efficacy and safety of triple combination therapy with artesunate-amodiaquine-methylene blue for falciparum malaria in children: a randomized controlled trial in Burkina Faso. J Infect Dis.

[70] Lefevre G, Looareesuwan S, Treeprasertsuk S, Krudsood S, Silachamroon U, Gathmann I, Mull R, Bakshi R (2001). A clinical and pharmacokinetic trial of six doses of artemether-lumefantrine for multidrug-resistant Plasmodium falciparum malaria in Thailand. Am J Trop Med Hyg.

[71] Gansane A, Nebie I, Soulama I, Tiono A, Diarra A, Konate AT, Ouedraogo A, Sirima BS (2009). Change of antimalarial first-line treatment in Burkina Faso in 2005. Bull Soc Pathol Exot.

[72] Bethell D, Se Y, Lon C, Tyner S, Saunders D, Sriwichai S, Darapiseth S, Teja-Isavadharm P, Khemawoot P, Schaecher K (2011). Artesunate dose escalation for the treatment of uncomplicated malaria in a region of reported artemisinin resistance: a randomized clinical trial. PLoS One.

[73] Nosten F, Luxemburger C, ter Kuile FO, Woodrow C, Eh JP, Chongsuphajaisiddhi T, White NJ (1994). Treatment of multidrug-resistant Plasmodium falciparum malaria with 3-day artesunate-mefloquine combination. J Infect Dis.

[74] Faye B, Kuete T, Kiki-Barro CP, Tine RC, Nkoa T, Ndiaye JL, Kakpo CA, Sylla K, El Menan H, Gaye O (2012). Multicentre study evaluating the non-inferiority of the new paediatric formulation of artesunate/amodiaquine versus artemether/lumefantrine for the management of uncomplicated Plasmodium falciparum malaria in children in Cameroon, Ivory Coast and Senegal. Malar J.

[75] Four Artemisinin-Based Combinations (4ABC) Study Group. A head-to-head comparison of four artemisinin-based combinations for treating uncomplicated malaria in African children: a randomized trial. PLoS Med. 2011;8(11):e1001119. 10.1371/journal.pmed.1001119 PMC3210754 22087077

[76] Das S, Chakraborty SP, Hati A, Roy S (2013). Malaria treatment failure with novel mutation in the Plasmodium falciparum dihydrofolate reductase (pfdhfr) gene in Kolkata, West Bengal, India. Int J Antimicrob Agents.

[77] van den Broek IV, Maung UA, Peters A, Liem L, Kamal M, Rahman M, Rahman MR, Bangali AM, Das S, Barends M (2005). Efficacy of chloroquine + sulfadoxine--pyrimethamine, mefloquine + artesunate and artemether + lumefantrine combination therapies to treat Plasmodium falciparum malaria in the Chittagong Hill Tracts, Bangladesh. Trans R Soc Trop Med Hyg.

[78] Gasasira AF, Dorsey G, Nzarubara B, Staedke SG, Nassali A, Rosenthal PJ, Kamya MR (2003). Comparative efficacy of aminoquinoline-antifolate combinations for the treatment of uncomplicated falciparum malaria in Kampala, Uganda. Am J Trop Med Hyg.

[79] Ngasala BE, Malmberg M, Carlsson AM, Ferreira PE, Petzold MG, Blessborn D, Bergqvist Y, Gil JP, Premji Z, Bjorkman A (2011). Efficacy and effectiveness of artemether-lumefantrine after initial and repeated treatment in children <5 years of age with acute uncomplicated Plasmodium falciparum malaria in rural Tanzania: a randomized trial. Clin Infect Dis.

[80] Carrasquilla G, Baron C, Monsell EM, Cousin M, Walter V, Lefevre G, Sander O, Fisher LM (2012). Randomized, prospective, three-arm study to confirm the auditory safety and efficacy of artemether-lumefantrine in Colombian patients with uncomplicated Plasmodium falciparum malaria. Am J Trop Med Hyg.

[81] Mayxay M, Khanthavong M, Lindegardh N, Keola S, Barends M, Pongvongsa T, Yapom R, Annerberg A, Phompida S, Phetsouvanh R (2004). Randomized comparison of chloroquine plus sulfadoxine-pyrimethamine versus artesunate plus mefloquine versus artemether-lumefantrine in the treatment of uncomplicated falciparum malaria in the Lao People's Democratic Republic. Clin Infect Dis.

[82] Adam I, Salah MT, Eltahir HG, Elhassan AH, Elmardi KA, Malik EM (2010). Dihydroartemisinin-piperaquine versus artemether-lumefantrine, in the treatment of uncomplicated Plasmodium falciparum malaria in central Sudan. Ann Trop Med Parasitol.

[83] Luxemburger C, ter Kuile FO, Nosten F, Dolan G, Bradol JH, Phaipun L, Chongsuphajaisiddhi T, White NJ (1994). Single day mefloquine-artesunate combination in the treatment of multi-drug resistant falciparum malaria. Trans R Soc Trop Med Hyg.

[84] Zongo I, Dorsey G, Rouamba N, Dokomajilar C, Lankoande M, Ouedraogo JB, Rosenthal PJ (2005). Amodiaquine, sulfadoxine-pyrimethamine, and combination therapy for uncomplicated falciparum malaria: a randomized controlled trial from Burkina Faso. Am J Trop Med Hyg.

[85] Bassat Q, Mulenga M, Tinto H, Piola P, Borrmann S, Menendez C, Nambozi M, Valea I, Nabasumba C, Sasi P (2009). Dihydroartemisinin-piperaquine and artemether-lumefantrine for treating uncomplicated malaria in African children: a randomised, non-inferiority trial. PLoS One.

[86] Thwing JI, Odero CO, Odhiambo FO, Otieno KO, Kariuki S, Ord R, Roper C, McMorrow M, Vulule J, Slutsker L (2009). In-vivo efficacy of amodiaquine-artesunate in children with uncomplicated Plasmodium falciparum malaria in western Kenya. Tropical Med Int Health.

[87] Ogutu BR, Onyango KO, Koskei N, Omondi EK, Ongecha JM, Otieno GA, Obonyo C, Otieno L, Eyase F, Johnson JD (2014). Efficacy and safety of artemether-lumefantrine and dihydroartemisinin-piperaquine in the treatment of uncomplicated Plasmodium falciparum malaria in Kenyan children aged less than five years: results of an open-label, randomized, single-centre study. Malar J.

[88] Djimde AA, Fofana B, Sagara I, Sidibe B, Toure S, Dembele D, Dama S, Ouologuem D, Dicko A, Doumbo OK (2008). Efficacy, safety, and selection of molecular markers of drug resistance by two ACTs in Mali. Am J Trop Med Hyg.

[89] Carrara VI, Zwang J, Ashley EA, Price RN, Stepniewska K, Barends M, Brockman A, Anderson T, McGready R, Phaiphun L (2009). Changes in the treatment responses to artesunate-mefloquine on the northwestern border of Thailand during 13 years of continuous deployment. PLoS One.

[90] Achan J, Tibenderana JK, Kyabayinze D, Wabwire Mangen F, Kamya MR, Dorsey G, D'Alessandro U, Rosenthal PJ, Talisuna AO (2009). Effectiveness of quinine versus artemether-lumefantrine for treating uncomplicated falciparum malaria in Ugandan children: randomised trial. BMJ.

[91] Checchi F, Piola P, Kosack C, Ardizzoni E, Klarkowski D, Kwezi E, Priotto G, Balkan S, Bakyaita N, Brockman A (2004). Antimalarial efficacy of sulfadoxine-pyrimethamine, amodiaquine and a combination of chloroquine plus sulfadoxine-pyrimethamine in Bundi Bugyo, western Uganda. Tropical Med Int Health.

[92] Barnes KI, Durrheim DN, Little F, Jackson A, Mehta U, Allen E, Dlamini SS, Tsoka J, Bredenkamp B, Mthembu DJ (2005). Effect of artemether-lumefantrine policy and improved vector control on malaria burden in KwaZulu-Natal, South Africa. PLoS Med.

[93] Ojurongbe O, Lawal OA, Abiodun OO, Okeniyi JA, Oyeniyi AJ, Oyelami OA (2013). Efficacy of artemisinin combination therapy for the treatment of uncomplicated falciparum malaria in Nigerian children. J Infect Dev Ctries.

[94] Ramharter M, Oyakhirome S, Klein Klouwenberg P, Adegnika AA, Agnandji ST, Missinou MA, Matsiegui PB, Mordmuller B, Borrmann S, Kun JF (2005). Artesunate-clindamycin versus quinine-clindamycin in the treatment of Plasmodium falciparum malaria: a randomized controlled trial. Clin Infect Dis.

[95] Price R, Luxemburger C, van Vugt M, Nosten F, Kham A, Simpson J, Looareesuwan S, Chongsuphajaisiddhi T, White NJ (1998). Artesunate and mefloquine in the treatment of uncomplicated multidrug-resistant hyperparasitaemic falciparum malaria. Trans R Soc Trop Med Hyg.

[96] Sirima SB, Tiono AB, Gansane A, Diarra A, Ouedraogo A, Konate AT, Kiechel JR, Morgan CC, Olliaro PL, Taylor WR (2009). The efficacy and safety of a new fixed-dose combination of amodiaquine and artesunate in young African children with acute uncomplicated Plasmodium falciparum. Malar J.

[97] Smithuis F, Kyaw MK, Phe O, Aye KZ, Htet L, Barends M, Lindegardh N, Singtoroj T, Ashley E, Lwin S (2006). Efficacy and effectiveness of dihydroartemisinin-piperaquine versus artesunate-mefloquine in falciparum malaria: an open-label randomised comparison. Lancet.

[98] Sutherland CJ, Ord R, Dunyo S, Jawara M, Drakeley CJ, Alexander N, Coleman R, Pinder M, Walraven G, Targett GA (2005). Reduction of malaria transmission to Anopheles mosquitoes with a six-dose regimen of co-artemether. PLoS Med.

[99] de Radigues X, Diallo KI, Diallo M, Ngwakum PA, Maiga H, Djimde A, Sacko M, Doumbo O, Guthmann JP (2006). Efficacy of chloroquine and sulfadoxine/pyrimethamine for the treatment of uncomplicated falciparum malaria in Koumantou, Mali. Trans R Soc Trop Med Hyg.

[100] Ashley EA, McGready R, Hutagalung R, Phaiphun L, Slight T, Proux S, Thwai KL, Barends M, Looareesuwan S, White NJ (2005). A randomized, controlled study of a simple, once-daily regimen of dihydroartemisinin-piperaquine for the treatment of uncomplicated, multidrug-resistant falciparum malaria. Clin Infect Dis.

[101] Tinto H, Diallo S, Zongo I, Guiraud I, Valea I, Kazienga A, Kpoda H, Sorgho H, Ouedraogo JB, Guiguemde TR (2014). Effectiveness of artesunate-amodiaquine vs. artemether-lumefantrine for the treatment of uncomplicated falciparum malaria in Nanoro, Burkina Faso: a non-inferiority randomised trial. Tropical Med Int Health.

[102] Yeka A, Dorsey G, Kamya MR, Talisuna A, Lugemwa M, Rwakimari JB, Staedke SG, Rosenthal PJ, Wabwire-Mangen F, Bukirwa H (2008). Artemether-lumefantrine versus dihydroartemisinin-piperaquine for treating uncomplicated malaria: a randomized trial to guide policy in Uganda. PLoS One.

[103] Premji Z, Umeh RE, Owusu-Agyei S, Esamai F, Ezedinachi EU, Oguche S, Borrmann S, Sowunmi A, Duparc S, Kirby PL (2009). Chlorproguanil-dapsone-artesunate versus artemether-lumefantrine: a randomized, double-blind phase III trial in African children and adolescents with uncomplicated Plasmodium falciparum malaria. PLoS One.

[104] Osorio L, Gonzalez I, Olliaro P, Taylor WR (2007). Artemisinin-based combination therapy for uncomplicated Plasmodium falciparum malaria in Colombia. Malar J.

[105] Zwang J, Olliaro P, Barennes H, Bonnet M, Brasseur P, Bukirwa H, Cohuet S, D'Alessandro U, Djimde A, Karema C (2009). Efficacy of artesunate-amodiaquine for treating uncomplicated falciparum malaria in sub-Saharan Africa: a multi-centre analysis. Malar J.

[106] Nhama A, Bassat Q, Enosse S, Nhacolo A, Mutemba R, Carvalho E, Naueia E, Sevene E, Guinovart C, Warsame M (2014). In vivo efficacy of artemether-lumefantrine and artesunate-amodiaquine for the treatment of uncomplicated falciparum malaria in children: a multisite, open-label, two-cohort, clinical trial in Mozambique. Malar J.

[107] Mayxay M, Khanthavong M, Chanthongthip O, Imwong M, Pongvongsa T, Hongvanthong B, Phompida S, Vanisaveth V, White NJ, Newton PN (2012). Efficacy of artemether-lumefantrine, the nationally-recommended artemisinin combination for the treatment of uncomplicated falciparum malaria, in southern Laos. Malar J.

[108] Grandesso F, Bachy C, Donam I, Ntambi J, Habimana J, D'Alessandro U, Maikere J, Vanlerberghe V, Kerah CH, Guthmann JP (2006). Efficacy of chloroquine, sulfadoxine-pyrimethamine and amodiaquine for treatment of uncomplicated Plasmodium falciparum malaria among children under five in Bongor and Koumra, Chad. Trans R Soc Trop Med Hyg.

[109] Mayxay M, Thongpraseuth V, Khanthavong M, Lindegardh N, Barends M, Keola S, Pongvongsa T, Phompida S, Phetsouvanh R, Stepniewska K (2006). An open, randomized comparison of artesunate plus mefloquine vs. dihydroartemisinin-piperaquine for the treatment of uncomplicated Plasmodium falciparum malaria in the Lao People's Democratic Republic (Laos). Tropical Med Int Health.

[110] Bell DJ, Wootton D, Mukaka M, Montgomery J, Kayange N, Chimpeni P, Hughes DA, Molyneux ME, Ward SA, Winstanley PA (2009). Measurement of adherence, drug concentrations and the effectiveness of artemether-lumefantrine, chlorproguanil-dapsone or sulphadoxine-pyrimethamine in the treatment of uncomplicated malaria in Malawi. Malar J.

[111] Luxemburger C, Price RN, Nosten F, Ter Kuile FO, Chongsuphajaisiddhi T, White NJ (1996). Mefloquine in infants and young children. Ann Trop Paediatr.

[112] van den Broek I, Amsalu R, Balasegaram M, Hepple P, Alemu E, Hussein el B, Al-Faith M, Montgomery J, Checchi F (2005). Efficacy of two artemisinin combination therapies for uncomplicated falciparum malaria in children under 5 years, Malakal, Upper Nile, Sudan. Malar J.

[113] Sagara I, Diallo A, Kone M, Coulibaly M, Diawara SI, Guindo O, Maiga H, Niambele MB, Sissoko M, Dicko A (2008). A randomized trial of artesunate-mefloquine versus artemether-lumefantrine for treatment of uncomplicated Plasmodium falciparum malaria in Mali. Am J Trop Med Hyg.

[114] Barnes KI, Little F, Smith PJ, Evans A, Watkins WM, White NJ (2006). Sulfadoxine-pyrimethamine pharmacokinetics in malaria: pediatric dosing implications. Clin Pharmacol Ther.

[115] Mehta U, Durrheim D, Mabuza A, Blumberg L, Allen E, Barnes KI (2007). Malaria pharmacovigilance in Africa: lessons from a pilot project in Mpumalanga Province. South Africa Drug Saf.

[116] Rwagacondo CE, Karema C, Mugisha V, Erhart A, Dujardin JC, Van Overmeir C, Ringwald P, D'Alessandro U (2004). Is amodiaquine failing in Rwanda? Efficacy of amodiaquine alone and combined with artesunate in children with uncomplicated malaria. Tropical Med Int Health.

[117] Checchi F, Balkan S, Vonhm BT, Massaquoi M, Biberson P (2002). Eldin de Pecoulas P, Brasseur P, Guthmann JP: Efficacy of amodiaquine for uncomplicated Plasmodium falciparum malaria in Harper, Liberia. Trans R Soc Trop Med Hyg.

[118] Nji AM, Ali IM, Moyeh MN, Ngongang EO, Ekollo AM, Chedjou JP, Ndikum VN, Evehe MS, Froeschl G, Heumann C (2015). Randomized non-inferiority and safety trial of dihydroartemisin-piperaquine and artesunate-amodiaquine versus artemether-lumefantrine in the treatment of uncomplicated Plasmodium falciparum malaria in Cameroonian children. Malar J.

[119] Abuaku B, Duah N, Quaye L, Quashie N, Koram K (2012). Therapeutic efficacy of artemether-lumefantrine combination in the treatment of uncomplicated malaria among children under five years of age in three ecological zones in Ghana. Malar J.

[120] ter Kuile FO, Dolan G, Nosten F, Edstein MD, Luxemburger C, Phaipun L, Chongsuphajaisiddhi T, Webster HK, White NJ (1993). Halofantrine versus mefloquine in treatment of multidrug-resistant falciparum malaria. Lancet.

[121] Staedke SG, Mpimbaza A, Kamya MR, Nzarubara BK, Dorsey G, Rosenthal PJ (2004). Combination treatments for uncomplicated falciparum malaria in Kampala, Uganda: randomised clinical trial. Lancet.

[122] Ogutu B, Juma E, Obonyo C, Jullien V, Carn G, Vaillant M, Taylor WR, Kiechel JR (2014). Fixed dose artesunate amodiaquine - a phase IIb, randomized comparative trial with non-fixed artesunate amodiaquine. Malar J.

[123] Toure OA, Assi SB, N'Guessan TL, Adji GE, Ako AB, Brou MJ, Ehouman MF, Gnamien LA, Coulibaly MA, Coulibaly B (2014). Open-label, randomized, non-inferiority clinical trial of artesunate-amodiaquine versus artemether-lumefantrine fixed-dose combinations in children and adults with uncomplicated falciparum malaria in Cote d'Ivoire. Malar J.

[124] van den Broek I, Kitz C, Al Attas S, Libama F, Balasegaram M, Guthmann JP (2006). Efficacy of three artemisinin combination therapies for the treatment of uncomplicated Plasmodium falciparum malaria in the Republic of Congo. Malar J.

[125] Sirima SB, Tiono AB, Konate A, Diarra A, Castelli F, Pinoges L, Mugittu K, Taylor WR, Olliaros PL (2003). Efficacy of artesunate plus chloroquine for the treatment of uncomplicated malaria in children in Burkina Faso: a double-blind, randomized, controlled trial. Trans R Soc Trop Med Hyg.

[126] Fogg C, Twesigye R, Batwala V, Piola P, Nabasumba C, Kiguli J, Mutebi F, Hook C, Guillerm M, Moody A (2008). Assessment of three new parasite lactate dehydrogenase (pan-pLDH) tests for diagnosis of uncomplicated malaria. Trans R Soc Trop Med Hyg.

[127] Juma EA, Obonyo CO, Akhwale WS, Ogutu BR (2008). A randomized, open-label, comparative efficacy trial of artemether-lumefantrine suspension versus artemether-lumefantrine tablets for treatment of uncomplicated Plasmodium falciparum malaria in children in western Kenya. Malar J.

[128] Sagara I, Dicko A, Djimde A, Guindo O, Kone M, Tolo Y, Thera MA, Sogoba M, Fofana M, Ouattara A (2006). A randomized trial of artesunate-sulfamethoxypyrazine-pyrimethamine versus artemether-lumefantrine for the treatment of uncomplicated Plasmodium falciparum malaria in Mali. Am J Trop Med Hyg.

[129] Adjei GO, Kurtzhals JA, Rodrigues OP, Alifrangis M, Hoegberg LC, Kitcher ED, Badoe EV, Lamptey R, Goka BQ (2008). Amodiaquine-artesunate vs artemether-lumefantrine for uncomplicated malaria in Ghanaian children: a randomized efficacy and safety trial with one year follow-up. Malar J.

[130] Hasugian AR, Purba HL, Kenangalem E, Wuwung RM, Ebsworth EP, Maristela R, Penttinen PM, Laihad F, Anstey NM, Tjitra E (2007). Dihydroartemisinin-piperaquine versus artesunate-amodiaquine: superior efficacy and posttreatment prophylaxis against multidrug-resistant Plasmodium falciparum and Plasmodium vivax malaria. Clin Infect Dis.

[131] Espie E, Lima A, Atua B, Dhorda M, Flevaud L, Sompwe EM, Palma Urrutia PP, Guerin PJ (2012). Efficacy of fixed-dose combination artesunate-amodiaquine versus artemether-lumefantrine for uncomplicated childhood Plasmodium falciparum malaria in Democratic Republic of Congo: a randomized non-inferiority trial. Malar J.

[132] Valecha N, Phyo AP, Mayxay M, Newton PN, Krudsood S, Keomany S, Khanthavong M, Pongvongsa T, Ruangveerayuth R, Uthaisil C (2010). An open-label, randomised study of dihydroartemisinin-piperaquine versus artesunate-mefloquine for falciparum malaria in Asia. PLoS One.

[133] Bell DJ, Nyirongo SK, Mukaka M, Zijlstra EE, Plowe CV, Molyneux ME, Ward SA, Winstanley PA (2008). Sulfadoxine-pyrimethamine-based combinations for malaria: a randomised blinded trial to compare efficacy, safety and selection of resistance in Malawi. PLoS One.

[134] Sutanto I, Suprijanto S, Kosasih A, Dahlan MS, Syafruddin D, Kusriastuti R, Hawley WA, Lobo NF, Ter Kuile FO (2013). The effect of primaquine on gametocyte development and clearance in the treatment of uncomplicated falciparum malaria with dihydroartemisinin-piperaquine in South sumatra, Western indonesia: an open-label, randomized, controlled trial. Clin Infect Dis.

[135] Ratcliff A, Siswantoro H, Kenangalem E, Maristela R, Wuwung RM, Laihad F, Ebsworth EP, Anstey NM, Tjitra E, Price RN (2007). Two fixed-dose artemisinin combinations for drug-resistant falciparum and vivax malaria in Papua, Indonesia: an open-label randomised comparison. Lancet.

[136] Ndiaye JL, Faye B, Gueye A, Tine R, Ndiaye D, Tchania C, Ndiaye I, Barry A, Cisse B, Lameyre V (2011). Repeated treatment of recurrent uncomplicated Plasmodium falciparum malaria in Senegal with fixed-dose artesunate plus amodiaquine versus fixed-dose artemether plus lumefantrine: a randomized, open-label trial. Malar J.

[137] Nosten F, ter Kuile FO, Luxemburger C, Woodrow C, Kyle DE, Chongsuphajaisiddhi T, White NJ (1993). Cardiac effects of antimalarial treatment with halofantrine. Lancet.

[138] Sirima SB, Ogutu B, Lusingu JPA, Mtoro A, Mrango Z, Ouedraogo A, Yaro JB, Onyango KO, Gesase S, Mnkande E (2016). Comparison of artesunate-mefloquine and artemether-lumefantrine fixed-dose combinations for treatment of uncomplicated Plasmodium falciparum malaria in children younger than 5 years in sub-Saharan Africa: a randomised, multicentre, phase 4 trial. Lancet Infect Dis.

[139] Faye B, Offianan AT, Ndiaye JL, Tine RC, Toure W, Djoman K, Sylla K, Ndiaye PS, Penali L, Gaye O (2010). Efficacy and tolerability of artesunate-amodiaquine (Camoquin plus) versus artemether-lumefantrine (Coartem) against uncomplicated Plasmodium falciparum malaria: multisite trial in Senegal and Ivory Coast. Tropical Med Int Health.

[140] Toure OA, Penali LK, Yapi JD, Ako BA, Toure W, Djerea K, Gomez GO, Makaila O (2009). A comparative, randomized clinical trial of artemisinin/naphtoquine twice daily one day versus artemether/lumefantrine six doses regimen in children and adults with uncomplicated falciparum malaria in Cote d'Ivoire. Malar J.

[141] van Vugt M, Looareesuwan S, Wilairatana P, McGready R, Villegas L, Gathmann I, Mull R, Brockman A, White NJ, Nosten F (2000). Artemether-lumefantrine for the treatment of multidrug-resistant falciparum malaria. Trans R Soc Trop Med Hyg.

[142] Yeka A, Lameyre V, Afizi K, Fredrick M, Lukwago R, Kamya MR, Talisuna AO (2014). Efficacy and safety of fixed-dose artesunate-amodiaquine vs. artemether-lumefantrine for repeated treatment of uncomplicated malaria in Ugandan children. PLoS One.

[143] Toure OA, Kouame MG, Didier YJ, Berenger AA, Djerea K, Genevieve GO, Penali LK (2011). Artesunate/mefloquine paediatric formulation vs. artemether/lumefantrine for the treatment of uncomplicated Plasmodium falciparum in Anonkoua koute, Cote d'Ivoire. Tropical Med Int Health.

[144] Bonnet M, Roper C, Felix M, Coulibaly L, Kankolongo GM, Guthmann JP (2007). Efficacy of antimalarial treatment in Guinea: in vivo study of two artemisinin combination therapies in Dabola and molecular markers of resistance to sulphadoxine-pyrimethamine in N'Zerekore. Malar J.

[145] Maiga AW, Fofana B, Sagara I, Dembele D, Dara A, Traore OB, Toure S, Sanogo K, Dama S, Sidibe B (2012). No evidence of delayed parasite clearance after oral artesunate treatment of uncomplicated falciparum malaria in Mali. Am J Trop Med Hyg.

[146] Zongo I, Dorsey G, Rouamba N, Dokomajilar C, Sere Y, Rosenthal PJ, Ouedraogo JB (2007). Randomized comparison of amodiaquine plus sulfadoxine-pyrimethamine, artemether-lumefantrine, and dihydroartemisinin-piperaquine for the treatment of uncomplicated Plasmodium falciparum malaria in Burkina Faso. Clin Infect Dis.

[147] Ursing J, Kofoed PE, Rodrigues A, Blessborn D, Thoft-Nielsen R, Bjorkman A, Rombo L (2011). Similar efficacy and tolerability of double-dose chloroquine and artemether-lumefantrine for treatment of Plasmodium falciparum infection in Guinea-Bissau: a randomized trial. J Infect Dis.

[148] Martensson A, Ngasala B, Ursing J, Isabel Veiga M, Wiklund L, Membi C, Montgomery SM, Premji Z, Farnert A, Bjorkman A (2007). Influence of consecutive-day blood sampling on polymerase chain reaction-adjusted parasitological cure rates in an antimalarial-drug trial conducted in Tanzania. J Infect Dis.

[149] Ashley EA, Lwin KM, McGready R, Simon WH, Phaiphun L, Proux S, Wangseang N, Taylor W, Stepniewska K, Nawamaneerat W (2006). An open label randomized comparison of mefloquine-artesunate as separate tablets vs. a new co-formulated combination for the treatment of uncomplicated multidrug-resistant falciparum malaria in Thailand. Tropical Med Int Health.

[150] Kamya MR, Yeka A, Bukirwa H, Lugemwa M, Rwakimari JB, Staedke SG, Talisuna AO, Greenhouse B, Nosten F, Rosenthal PJ (2007). Artemether-lumefantrine versus dihydroartemisinin-piperaquine for treatment of malaria: a randomized trial. PLoS Clin Trials.

[151] Mayxay M, Khanthavong M, Chanthongthip O, Imwong M, Lee SJ, Stepniewska K, Soonthornsata B, Pongvongsa T, Phompida S, Hongvanthong B (2012). No evidence for spread of Plasmodium falciparum artemisinin resistance to Savannakhet Province, Southern Laos. Am J Trop Med Hyg.

[152] Pareek A, Chandurkar N, Srivastav V, Lakhani J, Karmakar PS, Basu S, Ray A, Pednekar S, Gupta PB, Suthar N (2013). Comparative evaluation of efficacy and safety of artesunate-lumefantrine vs. artemether-lumefantrine fixed-dose combination in the treatment of uncomplicated Plasmodium falciparum malaria. Tropical Med Int Health.

[153] Price R, van Vugt M, Nosten F, Luxemburger C, Brockman A, Phaipun L, Chongsuphajaisiddhi T, White N (1998). Artesunate versus artemether for the treatment of recrudescent multidrug-resistant falciparum malaria. Am J Trop Med Hyg.

[154] Barennes H, Nagot N, Valea I, Koussoube-Balima T, Ouedraogo A, Sanou T, Ye S (2004). A randomized trial of amodiaquine and artesunate alone and in combination for the treatment of uncomplicated falciparum malaria in children from Burkina Faso. Tropical Med Int Health.

[155] Karunajeewa HA, Mueller I, Senn M, Lin E, Law I, Gomorrai PS, Oa O, Griffin S, Kotab K, Suano P (2008). A trial of combination antimalarial therapies in children from Papua New Guinea. N Engl J Med.

[156] Schramm B, Valeh P, Baudin E, Mazinda CS, Smith R, Pinoges L, Dhorda M, Boum Y, Sundaygar T, Zolia YM (2013). Efficacy of artesunate-amodiaquine and artemether-lumefantrine fixed-dose combinations for the treatment of uncomplicated Plasmodium falciparum malaria among children aged six to 59 months in Nimba County, Liberia: an open-label randomized non-inferiority trial. Malar J.

[157] Shekalaghe S, Drakeley C, Gosling R, Ndaro A, van Meegeren M, Enevold A, Alifrangis M, Mosha F, Sauerwein R, Bousema T (2007). Primaquine clears submicroscopic Plasmodium falciparum gametocytes that persist after treatment with sulphadoxine-pyrimethamine and artesunate. PLoS One.

[158] Bousema JT, Schneider P, Gouagna LC, Drakeley CJ, Tostmann A, Houben R, Githure JI, Ord R, Sutherland CJ, Omar SA (2006). Moderate effect of artemisinin-based combination therapy on transmission of Plasmodium falciparum. J Infect Dis.

[159] Hodel EM, Kabanywanyi AM, Malila A, Zanolari B, Mercier T, Beck HP, Buclin T, Olliaro P, Decosterd LA, Genton B (2009). Residual antimalarials in malaria patients from Tanzania--implications on drug efficacy assessment and spread of parasite resistance. PLoS One.

[160] von Seidlein L, Milligan P, Pinder M, Bojang K, Anyalebechi C, Gosling R, Coleman R, Ude JI, Sadiq A, Duraisingh M (2000). Efficacy of artesunate plus pyrimethamine-sulphadoxine for uncomplicated malaria in Gambian children: a double-blind, randomised, controlled trial. Lancet.

[161] Ndounga M, Mayengue PI, Casimiro PN, Loumouamou D, Basco LK, Ntoumi F, Brasseur P (2013). Artesunate-amodiaquine efficacy in Congolese children with acute uncomplicated falciparum malaria in Brazzaville. Malar J.

[162] Priotto G, Kabakyenga J, Pinoges L, Ruiz A, Eriksson T, Coussement F, Ngambe T, Taylor WR, Perea W, Guthmann JP (2003). Artesunate and sulfadoxine-pyrimethamine combinations for the treatment of uncomplicated Plasmodium falciparum malaria in Uganda: a randomized, double-blind, placebo-controlled trial. Trans R Soc Trop Med Hyg.

[163] Yavo W, Faye B, Kuete T, Djohan V, Oga SA, Kassi RR, Diatta M, Ama MV, Tine R, Ndiaye JL (2011). Multicentric assessment of the efficacy and tolerability of dihydroartemisinin-piperaquine compared to artemether-lumefantrine in the treatment of uncomplicated Plasmodium falciparum malaria in sub-Saharan Africa. Malar J.

[164] Faye B, Ndiaye JL, Tine R, Sylla K, Gueye A, Lo AC, Gaye O (2010). A randomized trial of artesunate mefloquine versus artemether lumefantrine for the treatment of uncomplicated Plasmodium falciparum malaria in Senegalese children. Am J Trop Med Hyg.

[165] Shayo A, Mandara CI, Shahada F, Buza J, Lemnge MM, Ishengoma DS (2014). Therapeutic efficacy and safety of artemether-lumefantrine for the treatment of uncomplicated falciparum malaria in North-Eastern Tanzania. Malar J.

[166] Adjei GO, Goka BQ, Enweronu-Laryea CC, Rodrigues OP, Renner L, Sulley AM, Alifrangis M, Khalil I, Kurtzhals JA (2014). A randomized trial of artesunate-amodiaquine versus artemether-lumefantrine in Ghanaian paediatric sickle cell and non-sickle cell disease patients with acute uncomplicated malaria. Malar J.

[167] Hwang J, Alemayehu BH, Hoos D, Melaku Z, Tekleyohannes SG, Teshi T, Birhanu SG, Demeke L, Gobena K, Kassa M (2011). In vivo efficacy of artemether-lumefantrine against uncomplicated Plasmodium falciparum malaria in Central Ethiopia. Malar J.

[168] Bukirwa H, Yeka A, Kamya MR, Talisuna A, Banek K, Bakyaita N, Rwakimari JB, Rosenthal PJ, Wabwire-Mangen F, Dorsey G (2006). Artemisinin combination therapies for treatment of uncomplicated malaria in Uganda. PLoS Clin Trials.

[169] Smithuis F, van der Broek I, Katterman N, Kyaw MK, Brockman A, Lwin S, White NJ (2004). Optimising operational use of artesunate-mefloquine: a randomised comparison of four treatment regimens. Trans R Soc Trop Med Hyg.

[170] Guthmann JP, Cohuet S, Rigutto C, Fortes F, Saraiva N, Kiguli J, Kyomuhendo J, Francis M, Noel F, Mulemba M (2006). High efficacy of two artemisinin-based combinations (artesunate + amodiaquine and artemether + lumefantrine) in Caala, Central Angola. Am J Trop Med Hyg.

[171] Laman M, Moore BR, Benjamin JM, Yadi G, Bona C, Warrel J, Kattenberg JH, Koleala T, Manning L, Kasian B (2014). Artemisinin-naphthoquine versus artemether-lumefantrine for uncomplicated malaria in Papua New Guinean children: an open-label randomized trial. PLoS Med.

[172] Faucher JF, Aubouy A, Adeothy A, Cottrell G, Doritchamou J, Gourmel B, Houze P, Kossou H, Amedome H, Massougbodji A (2009). Comparison of sulfadoxine-pyrimethamine, unsupervised artemether-lumefantrine, and unsupervised artesunate-amodiaquine fixed-dose formulation for uncomplicated plasmodium falciparum malaria in Benin: a randomized effectiveness noninferiority trial. J Infect Dis.

[173] Mockenhaupt FP, Ehrhardt S, Dzisi SY, Teun Bousema J, Wassilew N, Schreiber J, Anemana SD, Cramer JP, Otchwemah RN, Sauerwein RW (2005). A randomized, placebo-controlled, double-blind trial on sulfadoxine-pyrimethamine alone or combined with artesunate or amodiaquine in uncomplicated malaria. Tropical Med Int Health.

[174] Bonnet M, Broek I, van Herp M, Urrutia PP, van Overmeir C, Kyomuhendo J, Ndosimao CN, Ashley E, Guthmann JP (2009). Varying efficacy of artesunate+amodiaquine and artesunate+sulphadoxine-pyrimethamine for the treatment of uncomplicated falciparum malaria in the Democratic Republic of Congo: a report of two in-vivo studies. Malar J.

[175] Tahar R, Almelli T, Debue C, Foumane Ngane V, Djaman Allico J, Whegang Youdom S, Basco LK (2014). Randomized trial of artesunate-amodiaquine, atovaquone-proguanil, and artesunate-atovaquone-proguanil for the treatment of uncomplicated falciparum malaria in children. J Infect Dis.

[176] Martensson A, Stromberg J, Sisowath C, Msellem MI, Gil JP, Montgomery SM, Olliaro P, Ali AS, Bjorkman A (2005). Efficacy of artesunate plus amodiaquine versus that of artemether-lumefantrine for the treatment of uncomplicated childhood Plasmodium falciparum malaria in Zanzibar, Tanzania. Clin Infect Dis.

[177] Hatz C, Soto J, Nothdurft HD, Zoller T, Weitzel T, Loutan L, Bricaire F, Gay F, Burchard GD, Andriano K (2008). Treatment of acute uncomplicated falciparum malaria with artemether-lumefantrine in nonimmune populations: a safety, efficacy, and pharmacokinetic study. Am J Trop Med Hyg.

[178] White NJ (2018). Anaemia and malaria. Malar J.

[179] Zwang J, D’Alessandro U, Ndiaye J-L, Djimdé AA, Dorsey G, Mårtensson AA, Karema C, Olliaro PL (2017). Haemoglobin changes and risk of anaemia following treatment for uncomplicated falciparum malaria in sub-Saharan Africa. BMC Infect Dis.

[180] Calis JC, Phiri KS, Faragher EB, Brabin BJ, Bates I, Cuevas LE, de Haan RJ, Phiri AI, Malange P, Khoka M (2008). Severe anemia in Malawian children. N Engl J Med.

[181] Kho S, Qotrunnada L, Leonardo L, Andries B, Wardani PAI, Fricot A, Henry B, Hardy D, Margyaningsih NI, Apriyanti D (2021). Hidden biomass of intact malaria parasites in the human spleen. N Engl J Med.

[182] Imwong M, Suwannasin K, Kunasol C, Sutawong K, Mayxay M, Rekol H, Smithuis FM, Hlaing TM, Tun KM, van der Pluijm RW (2017). The spread of artemisinin-resistant Plasmodium falciparum in the Greater Mekong subregion: a molecular epidemiology observational study. Lancet Infect Dis.

[183] Balikagala B, Fukuda N, Ikeda M, Katuro OT, Tachibana SI, Yamauchi M, Opio W, Emoto S, Anywar DA, Kimura E (2021). Evidence of Artemisinin-Resistant Malaria in Africa. N Engl J Med.

[184] Uwimana A, Umulisa N, Venkatesan M, Svigel SS, Zhou Z, Munyaneza T, Habimana RM, Rucogoza A, Moriarty LF, Sandford R (2021). Association of Plasmodium falciparum kelch13 R561H genotypes with delayed parasite clearance in Rwanda: an open-label, single-arm, multicentre, therapeutic efficacy study. Lancet Infect Dis.

[185] Stepniewska K, Ashley E, Lee SJ, Anstey N, Barnes KI, Binh TQ, D'Alessandro U, Day NP, de Vries PJ, Dorsey G (2010). In vivo parasitological measures of artemisinin susceptibility. J Infect Dis.

