# Supplementary material for: Haematological consequences of acute uncomplicated falciparum malaria: a WorldWide Antimalarial Resistance Network pooled analysis of individual patient data
Source: BMC Med. 2022 Mar 7;20:85. doi: 10.1186/s12916-022-02265-9 (PMC8900374; doi:10.1186/s12916-022-02265-9)
Supplement: Supplementary file 2 — Additional file 2: Figure S1. Describes study sites. Figure S2. Describes the relationship between haemoglobin on enrolment and continuous covariates. Figure S3. Describes the relationship between the predicted probability of moderately severe anaemia on day 3 and continuous covariates. Figure S4. Describes the relationship between the predicted probability of a large fractional fall in haemoglobin on day 7 and continuous covariates. [file 12916_2022_2265_MOESM2_ESM.pdf]

Figure S1: Study sites for clinical trials

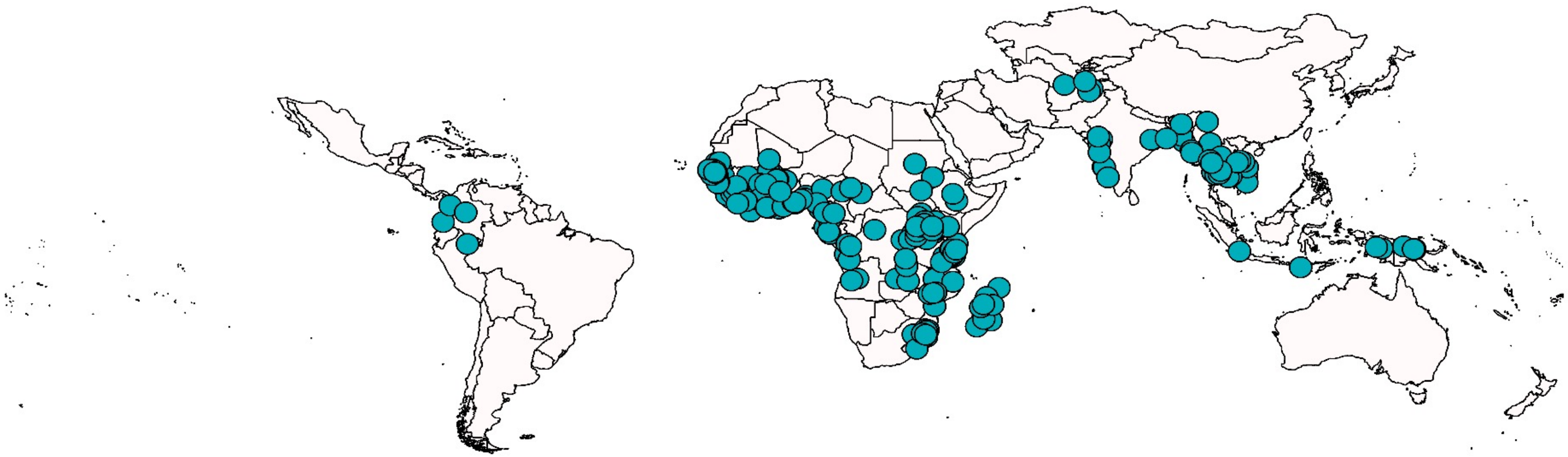

Figure S2: Relationship between haemoglobin on enrolment and continuous covariates

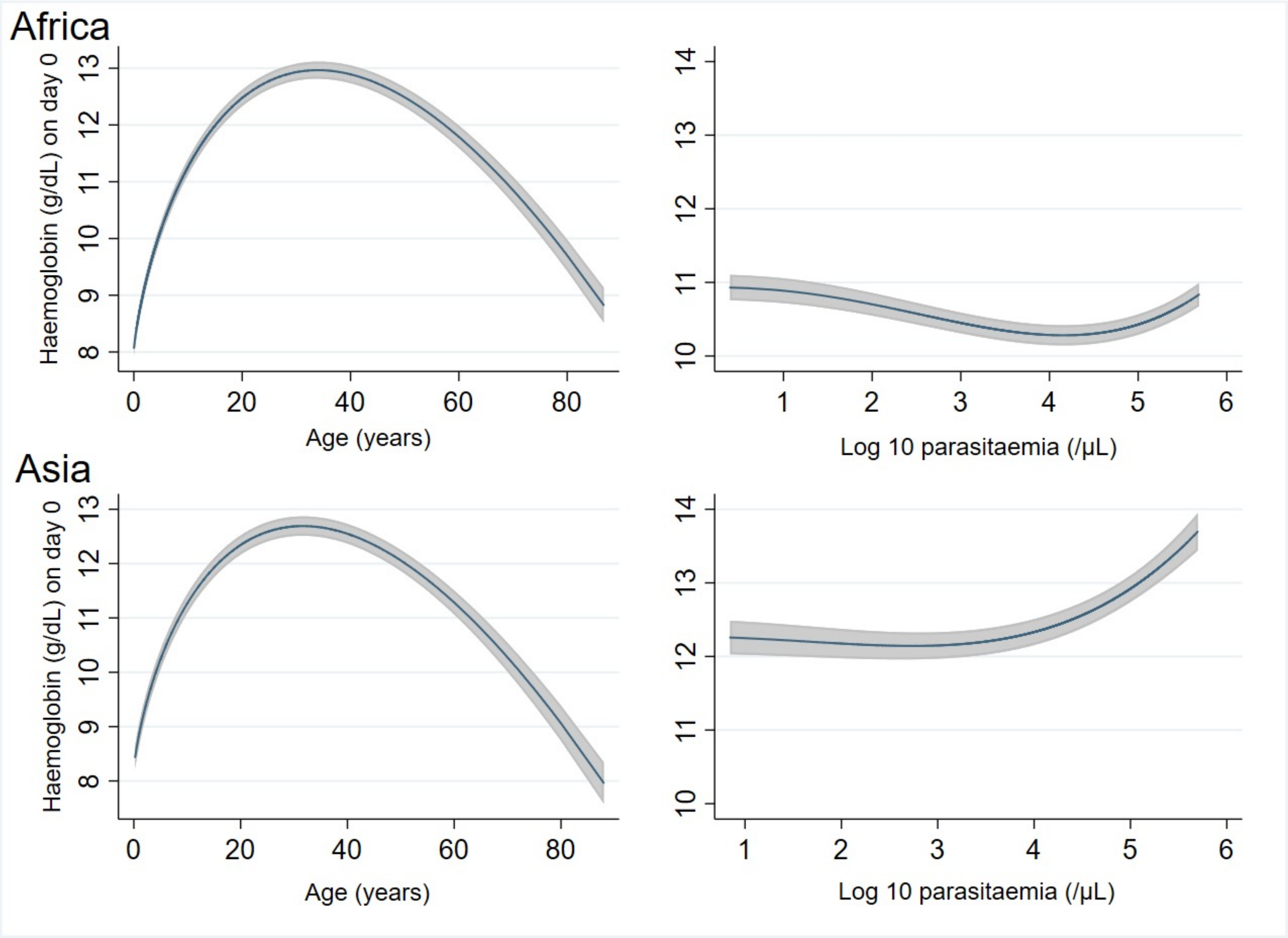

Results are generated from the final multivariable models and are adjusted for mean values of other covariates (age, baseline parasitaemia, sex and fever).

Figure S3: Relationship between predicted probabilities of moderately severe anaemia (haemoglobin <7g/dL) on day 3 and continuous covariates

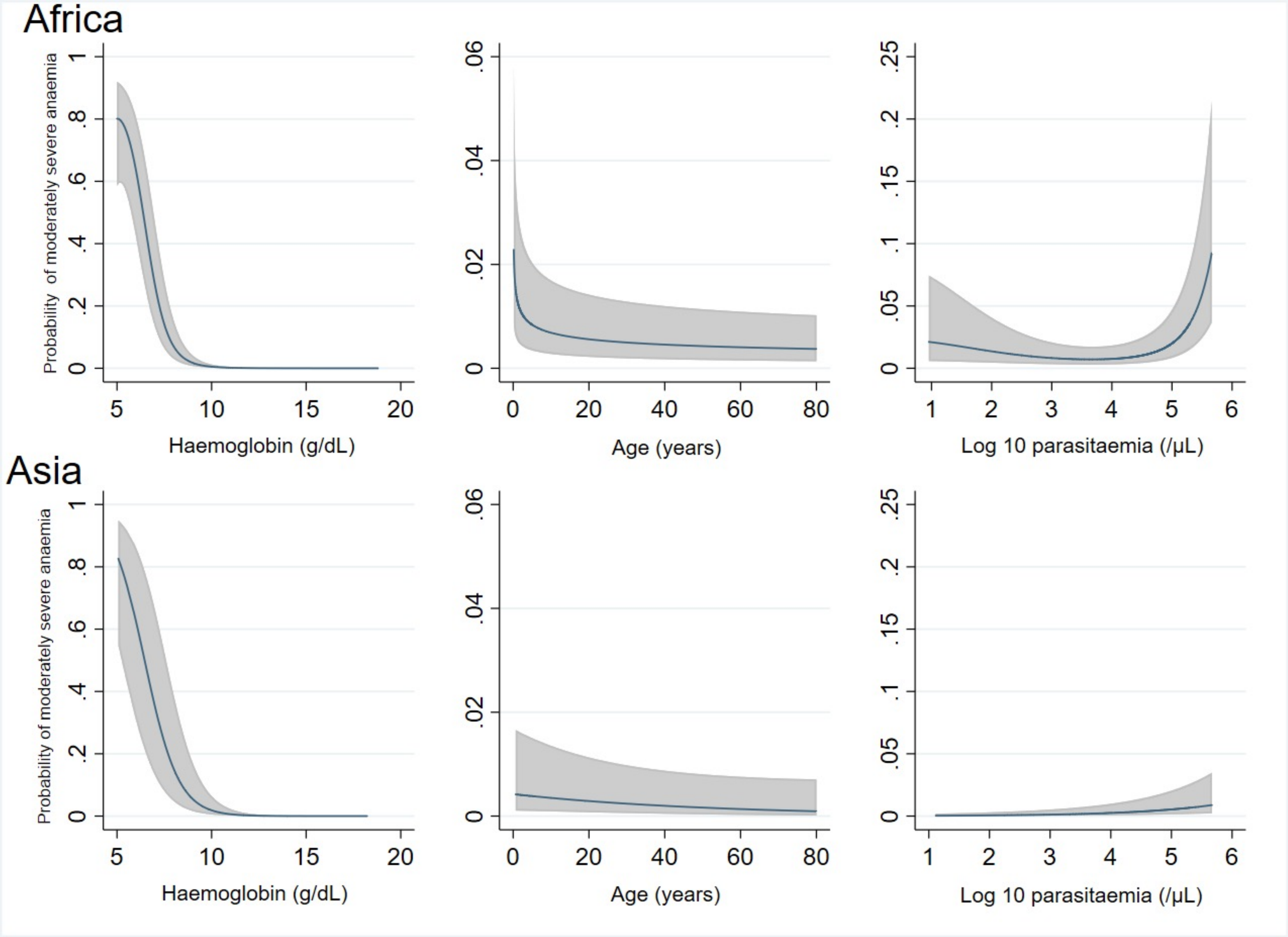

Results are generated from the final multivariable models and are adjusted for mean values of other covariates (severe anaemia at day0, baseline parasitaemia, sex, fever and treatment (artemisinin-based vs non-artemisinin-based)).

Figure S4: Relationship between predicted probabilities of a large fractional fall in haemoglobin on day 7 (≥25%) and continuous covariates

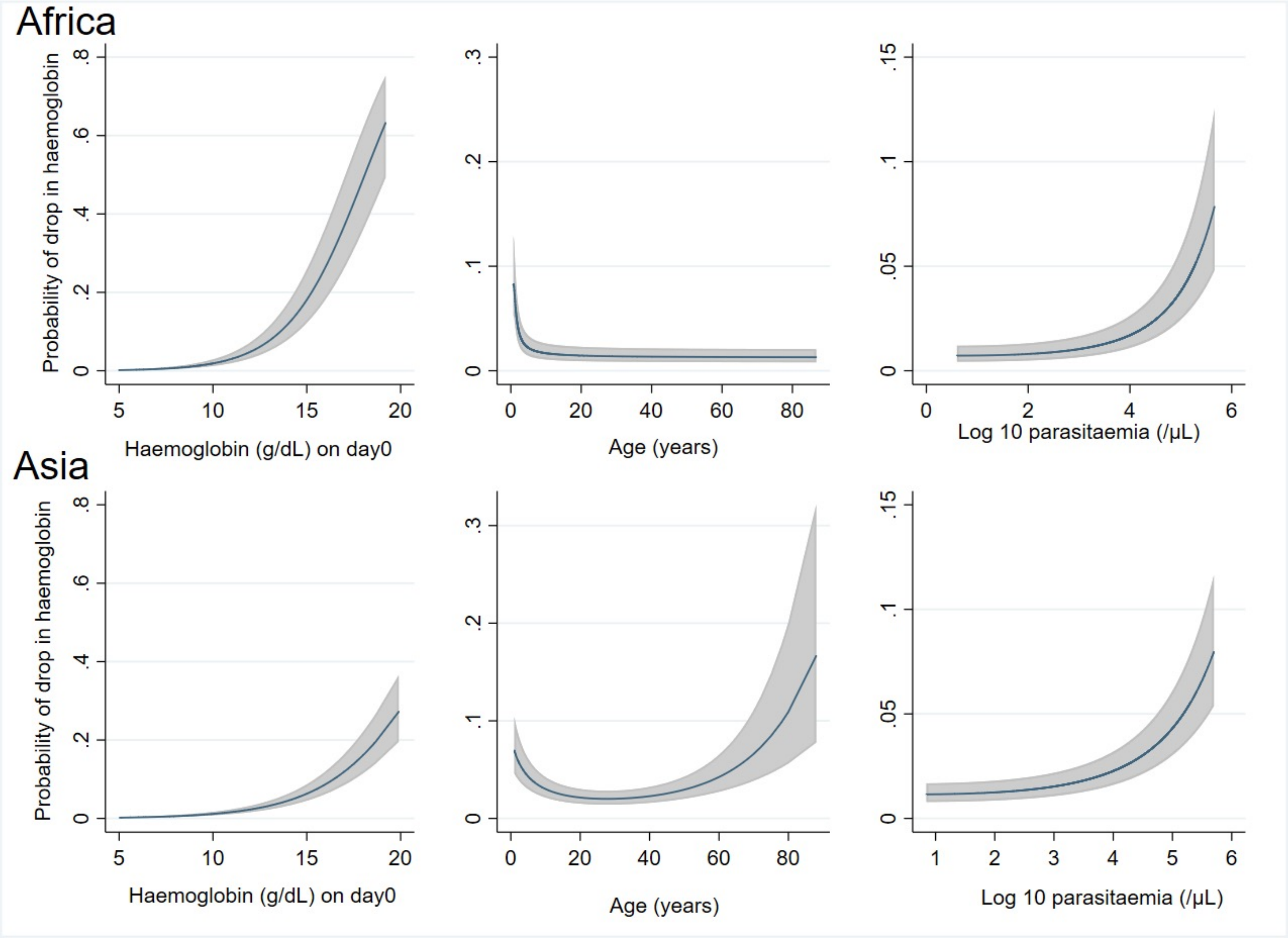

Results are generated from the final multivariable models and are adjusted for mean values of other covariates (haemoglobin at day 0, age, baseline parasitaemia, sex, fever, treatment (artemisinin-based vs non-artemisinin-based) and mixed infection (Asia only)). The model was restricted to children aged >0.75 years due to instability at the extremes of the data.
