# Supplementary material for: Haematological consequences of acute uncomplicated falciparum malaria: a WorldWide Antimalarial Resistance Network pooled analysis of individual patient data
Source: BMC Med. 2022 Mar 7;20:85. doi: 10.1186/s12916-022-02265-9 (PMC8900374; doi:10.1186/s12916-022-02265-9)
Supplement: Supplementary file 3 — Additional file 3: Table S3. Describes the overview of antimalarial treatments. Table S4. Describes the risk factors for moderately severe anaemia at enrolment (univariable logistic regression). Table S5. Describes the risk factors for moderately severe anaemia at enrolment (multivariable logistic regression). Table S6. Describes the risk factors for moderately severe anaemia at day 7 (univariable logistic regression). Table S7. Describes the risk factors for moderately severe anaemia at day 3 (univariable logistic regression). Table S8. Describes the risk factors for moderately severe anaemia at day 3 (multivariable logistic regression). Table S9. Describes the risk factors for a large fractional fall in haemoglobin by day 7. Table S10. Describes the sensitivity analysis for risk factors for moderately severe anaemia at enrolment (multivariable logistic regression). Table S11. Describes the sensitivity analysis for risk factors for moderately severe anaemia at day 7 (multivariable logistic regression). [file 12916_2022_2265_MOESM3_ESM.pdf]

Table S3: Overview of treatment

Treatment group	Number of patients (%) Africa (N=50,758)	Asia (N=18,446)	S America (N=916)
AL	14,966 (29.5%)	3,188 (17.3%)	205 (22.4%)
ASAQ	9,964 (19.6%)	531 (2.9%)	41 (4.5%)
ASAQ fixed dose combination	4,445 (44.6%)	426 (80.2%)	41 (100%)
ASAQ non- fixed dose combination	5,249 (52.7%)	105 (19.8%)	
ASAQ co-blister	270 (2.7%)		
ASMQ	1,363 (2.7%)	6,088 (33%)	313 (34.2%)
DP	5,205 (10.3%)	2730 (14.8%)	262 (28.6%)
Other artemisinin-based treatment[1]	5,325 (10.5%)	3,549 (19.2%)	0 (0%)
Non-artemisinin-based treatment[2]	13,935 (27.5%)	2,360 (12.8%)	95 (10.4%)

[1] Other artemisinin-based therapy includes artesunate monotherapy for 3, 5 or 7 days, artesunate-sulfadoxine-pyrimethamine, artesunate monotherapy followed by ACT, chlorproguanil-dapsone-artesunate, and dihydroartemisinin-piperaquine-trimethoprim; [2] Non-artemisinin-based therapy includes treatments with chloroquine, quinine, halofantrine, atovaquone-proguanil, amodiaquine, sulfadoxine-pyrimethamine, and mefloquine

Table S4: Risk factors for moderately severe anaemia (Hb <7g/dL) at enrolment: univariable logistic regression

	Africa			Asia		
**Parameter**	% (Number with moderately severe anaemia/N*) (%)	OR (95% CI)	P-value	% (Number with moderately severe anaemia/N*)	OR (95% CI)	P-value
Age (years)	8.4% (4,284/50,859)	0.74 (0.70-0.78)	<0.001	3.3% (606/18,451)	0.93 (0.91-0.95)	<0.001
Age Group						
<1 year	19.6% (892/4,562)	35.14 (23.05-53.57)	<0.001	7.5 % (5/67)	5.32 (2.12-13.33)	<0.001
1- 4 years	10.1% (3,142/31,225)	16.18 (10.50-24.93)	<0.001	12.3 % (254/2,071)	9.22 (6.62-12.86)	<0.001
5-11 years	2.3% (209/9,103)	3.40 (2.26-5.11)	<0.001	3.8 % (171/4,524)	2.59 (1.79-3.75)	<0.001
≥12 years	0.7% (41/5,969)	Reference		1.5 % (176/11,789)	Reference	
Sex[1]						
Female	8.3% (2,027/24,432)	0.97 (0.91-1.04)	0.366	4.1 % (290/7,054)	1.50 (1.23-1.84)	<0.001
Male	8.5% (2,181/25,566)	Reference		2.8 % (316/11,397)	Reference	
Log10 Parasitaemia	8.4% (4,284/50,859)	1.05 (0.90-1.24)	0.520	3.3% (606/18,451)	0.99 (0.83-1.19)	0.933
High parasitaemia[2]						
Yes	7.3% (380/5,200)	0.84 (0.73-0.97)	0.020	2.5 % (38/1,548)	0.72 (0.40-1.31)	0.283
No	8.6% (3,904/45,659)	Reference		3.4 % (568/16,903)	Reference	
Fever[3]						
Yes	8.9% (2,882/32,266)	1.30 (1.06-1.60)	0.013	3.0 % (269/8,937)	0.79 (0.66-0.94)	0.009
No	7.0% (1,174/16,716)	Reference		3.8 % (286/7,546)	Reference	
Presence of Gametocytaemia[4]						
Yes	14.5% (340/2,339)	2.47 (1.94-3.15)	<0.001	10.7 % (164/1,530)	5.19 (3.72-7.25)	<0.001
No	6.4% (1,681/26,114)	Reference		2.3 % (275/12,167)	Reference	
Underweight (WAZ<-2)[5]						
Yes	15.2% (941/6,205)	1.60 (1.41-1.81)	<0.001	13.3 % (104/781)	1.15 (0.83-1.58)	0.402
No	10.1% (2,701/26,843)	Reference		11.8 % (144/1,220)	Reference	
Mixed Infection						
Yes	0% (0/0)			3.7 % (43/1,151)	1.15 (0.48-2.75)	0.747
No	8.4% (4284/50,859)			3.3% (563/17,300)	Reference	
Transmission intensity area						
Low	7.4% (1,168/15,736)	0.75 (0.49-1.16)	0.201	3.1% (548/17,890)	0.27 (0.20-0.38)	<0.001
Moderate	7.9% (1,218/15,357)	0.81 (0.57-1.15)	0.234	10.3% (58/561)	Reference	
High	9.6% (1,898/19,766)	Reference		0% (0/0)		

*N = number of patients for each variable/levels of factors; OR = odds ratio; Total number of patients enrolled in Africa: 50,859 or Asia: 18,451; [1]Data on patients sex were only available for 49,998 patients from Africa; [2] Parasitaemia >100,000 /μL; [3]Data on baseline fever (Temperature>37.5°C) were only available for 48,982 patients from Africa and 16,483 patients from Asia; [4]Data on baseline gametocytes were only available for 28,453 patients from Africa or 13,697 patients from Asia; [5]Only evaluated in children <5 years; WAZ– weight-for-age Z-score; Data on weight-for-age Z-score was available for 33,048 patients from Africa and 2,001 patients from Asia.

Table S5: Independent risk factors for moderately severe anaemia (Hb <7g/dL) at enrolment: multivariable logistic regression

	Africa			Asia		
**Parameter**	% (Number with moderately severe anaemia/N*) n/N (%)	AOR (95% CI)	P-value	% (Number with moderately severe anaemia/N)	AOR (95% CI)	P-value
Age group						
<1 year	20.0% (833/4,162)	39.54 (26.61-58.75)	<0.001	7.9% (5/63)	6.13 (2.59-14.48)	<0.001
1-4 years	9.9% (2,921/29,538)	17.60 (11.73-26.41)	<0.001	12.9% (235/1,820)	10.23 (7.13-14.68)	<0.001
5-11 years	2.3% (192/8,493)	3.69 (2.39-5.68)	<0.001	3.8% (155/4,034)	2.69 (1.84-3.95)	<0.001
≥12 years	0.6% (37/5,936)	Reference		1.5% (160/10,566)	Reference	
Sex						
Female	8.2% (1,922/23,563)	0.99 (0.93-1.06)	0.781	4.3% (269/6,310)	1.26 (1.04-1.53)	0.017
Male	8.4% (2,061/24,566)	Reference		2.8% (286/10,173)	Reference	
Fever						
Yes	9.0% (2,835/31,624)	1.13 (0.97-1.32)	0.119	3.0% (269/8,937)	0.68 (0.57-0.82)	<0.001
No	7.0% (1,148/16,505)	Reference		3.8% (286/7,546)	Reference	
High parasitaemia[1]						
Yes	(7.3%) 359/4,930	0.73 (0.64-0.83)	<0.001	2.5% (34/1,377)	0.52 (0.33-0.82)	0.005
No	(8.4%) 3,624/43,199	Reference		3.5% (521/15,106)	Reference	

*N = number of patients for each variable/levels of factors; AOR = adjusted odds ratio; [1] Parasitaemia >100,000 /μL

Table S6: Risk factors for moderately severe anaemia (Hb <7g/dL) at day 7: univariable logistic regression

	Africa			Asia		
Parameter	% (Number with moderately severe anaemia/N*)	OR (95% CI)	P-value	% (Number with moderately severe anaemia/N*)	OR (95% CI)	P-value
Haemoglobin at day 0	5.7% (987/17,264)	0.38 (0.35-0.41)	<0.001	2.3% (254/11,064)	0.43 (0.39-0.47)	<0.001
Moderately severe anaemia at day 0						
Yes	42.5% (395/930)	19.63 (15.23-25.30)	<0.001	32.6 % (86/264)	30.58 (20.61-45.36)	<0.001
No	3.6% (592/16,334)	Reference		1.6% (168/10,800)	Reference	
Age (years)	5.7% (987/17,264)	0.81 (0.75-0.87)	<0.001	2.3% (254/11,064)	0.97 (0.95-0.99)	0.001
Age Group						
<1 year	16.8% (177/1,053)	27.81 (14.81-52.22)	<0.001	8.6% (3/35)	7.49 (2.94-19.09)	<0.001
1- 4 years	6.5% (675/10,402)	9.55 (5.23-17.46)	<0.001	6.9% (70/1,016)	5.91 (4.07-8.59)	<0.001
5-11 years	3.8% (115/3,036)	5.42 (2.93-10.02)	<0.001	3.5% (89/2,569)	2.87 (1.92-4.28)	<0.001
≥12 years	0.7% (20/2,773)	Reference		1.2% (92/7,444)	Reference	
Sex[1]						
Female	5.1% (427/8,306)	0.83 (0.73-0.94)	0.004	3.0% (130/4,301)	1.67 (1.30-2.15)	<0.001
Male	6.2% (545/8,854)	Reference		1.8% (124/6,763)	Reference	
Log10 Parasitaemia	5.7% (987/17,264)	1.62 (1.27-2.06)	<0.001	2.3% (254/11,064)	1.30 (1.10-1.55)	0.003
High parasitaemia[2]						
Yes	9.1% (154/1,700)	1.76 (1.37-2.27)	<0.001	3.8% (39/1,039)	1.78 (1.13-2.81)	0.014
No	5.4% (833/15,564)	Reference		2.1% (215/10,025)	Reference	
Fever[3]						
Yes	7.0% (710/10,179)	1.91 (1.39-2.62)	<0.001	2.7% (148/5,578)	1.32 (1.00-1.75)	0.053
No	3.8% (251/6,651)	Reference		2.0% (92/4,553)	Reference	
Presence of gametocytaemia[4]						
Yes	5.5% (61/1,107)	0.99 (0.66-1.48)	0.969	6.1% (48/783)	3.20 (2.09-4.91)	<0.001
No	5.6% (720/12,968)	Reference		2.0% (171/8,562)	Reference	
Underweight (WAZ<-2)[5]						
Yes	9.2% (192/2,098)	1.33 (1.01-1.76)	0.040	7.9% (22/278)	1.14 (0.63-2.09)	0.662

No	7.0% (650/9,263)	Reference		7.0% (45/644)	Reference	
Mixed Infection						
Yes	0% (0/0)			1.5% (11/724)	0.64 (0.30-1.37)	0.251
No	5.7% (987/17,264)			2.4% (243/10,340)	Reference	
Transmission intensity area						
Low	5.7% (407/7,099)	0.99 (0.59-1.66)	0.963	2.3% (249/11038)	10.32 (8.15-13.06)	<0.001
Moderate	5.5% (203/3,665)	0.95 (0.58-1.55)	0.845	19.2% (5/26)	Reference	
High	5.8% (377/6,500).	Reference		0% (0/0)		
Day 1 positivity[6]						
Yes	6.7% (627/9,377)	1.38 (1.02-1.85)	0.034	2.9% (183/6,356)	1.80 (1.24-2.61)	0.002
No	4.9% (268/5,420)	Reference		1.6% (59/3,645)	Reference	
Day 2 positivity[7]						
Yes	9.9% (168/1,690)	2.00 (1.41-2.83)	<0.001	3.4% (54/1,600)	1.68 (1.13-2.48)	0.010
No	5.2% (751/14,355)	Reference		2.0% (167/8,180)	Reference	
Day 3 positivity[8]						
Yes	10.7% (46/429)	2.10 (1.32-3.35)	0.002	2.9% (11/382)	1.36 (0.68-2.71)	0.386
No	5.4% (874/16,166)	Reference		2.1% (199/9,310)	Reference	
Delay in parasite clearance[9]						
Yes	9.2% (144/1,563)	1.71 (1.26-2.33)	0.001	2.8% (37/1,339)	1.29 (0.87-1.92)	0.206
No	5.6% (696/12,441)	Reference		2.2% (164/7,621)	Reference	
Clearance time						
Clearance between day 1 and day 2	6.1% (428/7,021)	1.25 (0.94-1.66)	0.125	2.6% (105/3,976)	1.65 (1.15-2.36)	0.007
Clearance between day 2 and day 3	8.3% (100/1,201)	1.75 (1.16-2.62)	0.007	2.7% (26/964)	1.68 (1.00-2.83)	0.049
Clearance after day 3	12.2% (44/362)	2.66 (1.57-4.51)	<0.001	2.9% (11/375)	1.84 (0.89-3.80)	0.101
Clearance between day 0 and day 1	4.9% (268/5,420)	Reference		1.6% (59/3,645)	Reference	
Treatment						
Artemisinin-based	5.6% (881/15,736)	0.76 (0.39-1.47)	0.411	2.5% (238/9,669)	2.18 (1.29-3.66)	0.004
Non-artemisinin-based	7.3% (106/1,462)	Reference		1.2% (16/1,395)	Reference	

*N = number of patients for each variable/levels of factors; OR = odds ratio; Total number of patients from Africa: 17,264 or Asia: 11,064; [1]Data on patients sex were only available for 17,160 patients from Africa; [2] Parasitaemia >100,000 /µL; [3]Data on baseline fever (Temperature>37.5°C) were only available for 16,830 patients from Africa and 10,131 patients from Asia; [4]Data on baseline gametocytes were only available for 14,075 patients from Africa and 9,345 patients from Asia; [5] Only evaluated in children <5 years; WAZ – weight-for-age Z-score; Data on weight-for-age Z-score were available for 11,361 patients from Africa and 922 patients from Asia; [6]Data on Day 1 positivity status were only available for 14,797 patients from Africa and 10,001 patients from Asia; [7]Data on Day 2 positivity status were only available for 16,045 patients from Africa and 9,780 patients from Asia; [8]Data on Day 3 positivity status were only available for 16,595 patients from Africa and 9,692 patients from Asia; [9]Clearance on day 3 or later.

Table S7: Risk factors for moderately severe anaemia (Hb< 7g/dL) at day 3: univariable logistic regression

	Africa			Asia		
Parameter	% (Number with moderately severe anaemia/N*)	OR (95% CI)	P-value	% (Number with moderately severe anaemia/N*)	OR (95% CI)	P-value
Haemoglobin at day 0	9.6% (987/10,278)	0.26 (0.23-0.31)	<0.001	6.5% (142/2,182)	0.33 (0.27-0.41)	<0.001
Moderately severe anaemia at day 0						
Yes	65.1% (393/604)	28.47 (20.48-39.59)	<0.001	78.6% (45/58)	72.34 (33.72-155.17)	<0.001
No	6.1% (594/9,674)	Reference		4.6% (97/2,124)	Reference	
Age (years)	9.6% (987/10,278)	0.79 (0.71-0.89)	<0.001	6.5% (142/2,182)	0.91 (0.87-0.95)	<0.001
Age group						
<1 year	19.5% (138/706)	21.38 (9.81-46.58)	<0.001	0.0% (0/3)	Excluded from model	
1- 4 years	10.0% (775/7,777)	9.74 (4.47-21.22)	<0.001	20.2% (71/351)	10.17 (5.36-19.31)	<0.001
5-11 years	5.7% (67/1,172)	5.34 (2.50-11.40)	<0.001	9.3% (36/389)	4.09 (1.89-8.83)	<0.001
≥12 years	1.1% (7/623)	Reference		2.4% (35/1,439)	Reference	
Sex[1]						
Female	8.8% (438/4,954)	0.86 (0.76-0.97)	0.014	8.3% (69/832)	1.58 (0.92-2.71)	0.094
Male	10.1% (531/5,241)	Reference		5.4% (73/1,350)	Reference	
Log10 Parasitaemia	9.6% (987/10,278)	1.54 (1.24-1.92)	0.001	6.5% (142/2,182)	1.41 (1.15-1.74)	0.001
High parasitaemia[2]						
Yes	14.2% (168/1,181)	1.68 (1.36-2.06)	<0.001	8.8% (21/238)	1.46 (0.98-2.16)	0.061
No	9.0% (819/9,097)	Reference		6.2% (121/1,944)	Reference	
Fever[3]						
Yes	10.7% (663/6,184)	1.42 (1.12-1.80)	0.004	5.1% (47/925)	0.69 (0.48-0.99)	0.044
No	7.8% (296/3,787)	Reference		7.2% (40/553)	Reference	
Presence of gametocytaemia[4]						
Yes	10.4% (91/879)	1.14 (0.71-1.81)	0.595	14.1% (33/234)	5.82 (2.21-15.31)	<0.001
No	9.2% (804/8,706)	Reference		2.7% (20/729)	Reference	
Underweight (WAZ<-2)[5]						
Yes	13.4% (217/1,620)	1.37 (1.13-1.65)	0.001	22.7% (15/66)	1.36 (0.97-1.91)	0.077

No	10.2% (691/6,798)	Reference		17.8% (29/163)	Reference	
Mixed Infection						
Yes	0.0% (0/0)			18.2% (2/11)	3.22 (1.86-5.58)	<0.001
No	9.6% (987/10,278)			6.4% (140/2,171)	Reference	
Transmission intensity area						
Low	9.2% (295/3200)	0.94 (0.56-1.59)	0.823	6.5% (142/2182)		
Moderate	9.9% (236/2391)	1.02 (0.66-1.56)	0.942	0.0% (0/0)		
High	9.7% (456/4687)	Reference		0.0% (0/0)		
Day 1 positivity[6]						
Yes	11.6% (661/5,717)	1.80 (1.44-2.25)	<0.001	7.2% (87/1,205)	1.70 (0.65-4.44)	0.282
No	6.8% (247/3,651)	Reference		4.4% (39/889)	Reference	
Day 2 positivity[7]						
Yes	16.3% (123/756)	1.97 (1.39-2.79)	<0.001	4.8% (23/476)	0.91 (0.36-2.27)	0.835
No	9.0% (769/8,568)	Reference		5.3% (81/1,528)	Reference	
Day 3 positivity[8]						
Yes	12.0% (14/117)	1.34 (0.91-1.98)	0.144	2.5% (5/198)	0.45 (0.11-1.82)	0.264
No	9.2% (898/9,753)	Reference		5.4% (97/1,789)	Reference	
Delay in parasite clearance[9]						
Yes	14.7% (105/712)	1.73 (1.31-2.29)	<0.001	4.7% (21/451)	0.88 (0.34-2.24)	0.786
No	9.1% (732/8,051)	Reference		5.3% (80/1,519)	Reference	
Clearance time						
Clearance between day 1 and day 2	11.0% (485/4,400)	1.71 (1.39-2.09)	<0.001	6.5% (41/630)	1.52 (0.81-2.85)	0.196
Clearance between day 2 and day 3	15.2% (96/631)	2.47 (1.71-3.58)	<0.001	6.3% (16/254)	1.47 (0.45-4.73)	0.523
Clearance after day 3	11.1% (9/81)	1.72 (0.95-3.13)	0.074	2.5% (5/197)	0.57 (0.11-2.86)	0.493
Clearance between day 0 and day 1	6.8% (247/3,651)	Reference		4.4% (39/889)	Reference	
Treatment						
Artemisinin-based	9.5% (962/10,101)	0.64 (0.15-2.76)	0.550	6.6% (137/2,076)	1.43 (0.82-2.49)	0.210
Non-artemisinin-based	14.1% (25/177)	Reference		4.7% (5/106)	Reference	

*N = number of patients for each variable/levels of factors; OR = odds ratio; Total number of patients from Africa: 10,278 or Asia: 2,182; [1]Data on patients sex were only available for 10,195 patients from Africa; [2] Parasitaemia >100,000 /µL; [3]Data on baseline fever (Temperature>37.5°C) were only available for 9,971 patients from Africa and 1,478 patients from Asia; [4]Data on baseline gametocytes were only available for 9,585 patients from Africa or 963 patients from Asia; [5] Only evaluated in children <5 years; WAZ – weight-for-age Z score; Data on weight-for-age Z-score were available for 8,418 patients from Africa and 229 patients from Asia; [6]Data on Day 1 positivity status were only available for 9,368 patients from Africa and 2,094 patients from Asia; [7]Data on Day 2 positivity status were only available for 9,324 patients from Africa and 2,004 patients from Asia; [8]Data on Day 3 positivity status were only available for 9,870 patients from Africa and 1,987 patients from Asia; [9] Clearance on day 3 or later.

Table S8: Independent risk factors for moderately severe anaemia (Hb< 7g/dL) at day 3: multivariable logistic regression

	Africa			Asia		
**Parameter**	% (Number with moderately severe anaemia/N*)	AOR (95% CI)	P-value	% (Number with moderately severe anaemia/N*)	AOR (95% CI)	P-value
Age group						
<1 year	19.7 % (138/701)	18.35 (8.88-37.93)	<0.001		Excluded from model	
1-4 years	9.9% (749/7,562)	10.18 (5.28-19.60)	<0.001	20.2% (71/351)	7.52 (4.03-14.01)	<0.001
5-11 years	5.0% (51/1,020)	6.27 (2.89-13.59)	<0.001	9.3% (36/389)	4.08 (2.07-8.03)	<0.001
≥12 years	0.8% (5/609)	Reference		2.4% (35/1,439)	Reference	
Sex						
Female	8.9% (426/4,803)	0.88 (0.75-1.03)	0.121	8.3% (69/831)	1.04 (0.70-1.56)	0.836
Male	10.2% (517/5,089)	Reference		5.4% (73/1,348)	Reference	
Fever						
Yes	10.6% (651/6,120)	1.47 (1.21-1.78)	<0.001		Not significant[2]	
No	7.7% (292/3,772)	Reference			Reference	
Moderately severe anaemia at day 0						
Yes	65.6% (383/584)	29.00 (20.52-40.99)	<0.001	77.6% (45/58)	53.79 (19.87-145.62)	<0.001
No	6.0% (560/9,308)	Reference		4.6% (97/2,121)	Reference	
High parasitaemia[1]						
Yes	14.2% (161/1,135)	2.16 (1.78-2.64)	<0.001	8.8% (21/238)	1.36 (0.84-2.19)	0.209
No	8.9% (782/8,757)	Reference		6.2% (121/1,941)	Reference	
Treatment						
Artemisinin-based	9.4% (918/9,716)	0.69 (0.32-1.49)	0.343	6.6% (137/2,073)	3.27 (2.42-4.42)	<0.001
Non-artemisinin-based	14.2% (25/176)	Reference		4.7% (5/106)	Reference	

*N = number of patients for each variable/levels of factors; AOR = adjusted odds ratio; [1] Parasitaemia >100,000 /µL; [2] Fever was not significant in multivariable model in Asia

Table S9: Independent risk factors for a large fractional fall in haemoglobin (≥25%) by day 7

Parameter	Africa % (Number with large fractional fall /N*)	Africa AOR (95% CI)	Africa P-value	Asia % (Number with large fractional fall /N*)	Asia AOR (95% CI)	Asia P-value
Haemoglobin at day 0	585/16,663 (3.5%)	1.52 (1.40-1.65)	<0.001	8.0% (809/10,131)	1.43 (1.35-1.52)	<0.001
Age group						
< 1 year	4.1% (43/1,045)	4.73 (2.39-9.35)	<0.001	9.7% (3/31)	3.43 (1.41-8.34)	0.007
1-4 years	3.3% (328/10,086)	2.48 (1.41-4.36)	0.002	10.0% (86/861)	2.91 (2.09-4.03)	<0.001
5-11 years	4.1% (115/2,826)	2.01 (1.22-3.31)	0.006	8.9% (209/2,341)	1.82 (1.38-2.38)	<0.001
≥12 years	3.7% (99/2,706)	Reference		7.4% (511/6,898)	Reference	
Sex						
Female	3.6% (287/8,058)	1.07 (0.91-1.27)	0.412	9.7% (378/3,884)	1.92 (1.58-2.34)	<0.0001
Male	3.5% (298/8,605)	Reference		6.9% (431/6,247)	Reference	
Fever						
Yes	4.1% (416/10,062)	1.63 (1.15-2.30)	0.006	9.9% (550/5,578)	1.57 (1.31-1.87)	<0.001
No	2.6% (169/6,601)	Reference		5.7% (259/4,553)	Reference	
High parasitaemia[1]						
Yes	6.8% (110/1,621)	2.15 (1.64-2.84)	<0.001	19.8% (178/900)	2.54 (2.11-3.05)	<0.001
No	3.2% (475/15,042)	Reference		6.8% (631/9,231)	Reference	
Mixed Infection						
Yes	0% (0/0)			6.0% (43/717)	0.64 (0.50-0.83)	0.001
No	3.5% (585/16,663)			8.1% (766/9,414)	Reference	
Treatment						
Artemisinin-based	3.3% (501/15,209)	0.59 (0.34-1.02)	0.059	8.0% (718/8,958)	0.94 (0.75-1.16)	0.558
Non-artemisinin-based	5.8% (84/1,454)	Reference		7.8% (91/1,173)	Reference	

*N = number of patients for each variable/levels of factors; AOR = adjusted odds ratio; [1] Parasitaemia >100,000 /µL

Table S10: Sensitivity analysis of independent risk factors for moderately severe anaemia (Hb <7g/dL) at enrolment after exclusion of studies with baseline cut-offs >5 g/dL: multivariable logistic regression

	Africa			Asia		
**Parameter**	% (Number with moderately severe anaemia/N*) n/N (%)	AOR (95% CI)	P-value	% (Number with moderately severe anaemia/N)	AOR (95% CI)	P-value
Age group						
<1 year	21.2% (815/3,837)	41.05 (27.44-61.42)	<0.001	8.2% (5/61)	6.07 (2.58-14.28)	<0.001
1-4 years	11.3% (2,808/24,903)	19.55 (12.92-29.57)	<0.001	13.1% (228/1,735)	9.96 (6.89-14.40)	<0.001
5-11 years	2.3% (188/8,019)	3.66 (2.35-5.69)	<0.001	4.0% (150/3,735)	2.70 (1.81-4.02)	<0.001
≥12 years	0.7% (36/5,484)	Reference		1.6% (156/9,839)	Reference	
Sex						
Female	8.9% (1,858/20,811)	0.98 (0.92-1.05)	0.581	4.5% (262/5,835)	1.28 (1.05-1.56)	0.014
Male	9.3% (1,989/21,432)	Reference		2.9% (277/9,535)	Reference	
Fever						
Yes	9.7% (2,762/28,371)	1.06 (0.90-1.24)	0.502	3.1% (262/8,393)	0.68 (0.56-0.83)	<0.001
No	7.8% (1,085/13,872)	Reference		4.0% (277/6,977)	Reference	
High parasitaemia[1]						
Yes	(8.1%) 345/4,261	0.73 (0.64-0.83)	<0.001	2.5% (34/1,372)	0.50 (0.32-0.79)	0.003
No	(9.2%) 3,502/37,982	Reference		3.6% (505/13,998)	Reference	

*N = number of patients for each variable/levels of factors; AOR = adjusted odds ratio; [1] Parasitaemia >100,000 /µL

Table S11: Sensitivity analysis of independent risk factors for moderately severe anaemia (Hb <7g/dL) at day 7 after exclusion of studies with baseline cut-offs >5 g/dL: multivariable logistic regression

Parameter	Africa % (Number with moderately severe anaemia/N)	Africa AOR (95% CI)	Africa P-value	Asia % (Number with moderately severe anaemia/N)	Asia AOR (95% CI)	Asia P-value
Age group						
<1 year	17.8% (134/755)	11.18 (5.81-21.51)	<0.001	9.7% (3/31)	6.58 (2.29-18.94)	<0.001
1-4 years	7.7% (480/6,208)	6.36 (3.42-11.83)	<0.001	7.4% (63/857)	2.82 (1.82-4.35)	<0.001
5-11 years	3.7% (99/2,677)	4.43 (2.29-8.56)	<0.001	3.7% (83/2,238)	2.37 (1.57-3.58)	<0.001
≥12 years	0.7% (18/2,622)	Reference		1.4% (91/6,567)	Reference	
Sex						
Female	5.5% (326/5,968)	0.81 (0.68-0.97)	0.019	3.3% (124/3,714)	1.52 (1.15-2.00)	0.003
Male	6.4% (405/6,294)	Reference		1.9% (116/5,979)	Reference	
Fever						
Yes	7.2% (547/7,564)	1.57 (1.18-2.07)	0.002	2.8% (148/5,384)	1.24 (0.91-1.69)	0.180
No	3.9% (184/4,698)	Reference		2.1% (92/4,309)	Reference	
Moderately severe anaemia						
Yes	42.6% (336/788)	15.70 (12.04-20.48)	<0.001	32.6% (78/239)	22.10 (13.85-35.29)	<0.001
No	3.4% (395/11,474)	Reference		1.7% (162/9,454)		
High parasitaemia[1]						
Yes	9.8% (106/1,087)	1.70 (1.25-2.30)	<0.001	3.9% (35/898)	1.52 (1.06-2.18)	0.021
No	5.6% (625/11,175)	Reference		2.3% (205/8,795)	Reference	
Mixed Infection						
Yes	0% (0/0)			1.5% (11/717)	0.42 (0.23-0.78)	0.005
No	6.0% (731/12,262)			2.6% (229/8,976)	Reference	
Treatment						
Artemisinin-based	5.8% (625/10,809)	1.01 (0.56-1.80)	0.981	2.6% (225/8,594)	2.05 (1.40-3.01)	<0.001
Non-artemisinin-based	7.3% (106/1,453)	Reference		1.4% (15/1,099)	Reference	

N = total number of evaluable patients; AOR = adjusted odds ratio; [1] Parasitaemia >100,000 /µL.
